# Efficacy of lifestyle interventions in the management of systemic lupus erythematosus: a systematic review of the literature

**DOI:** 10.1007/s00296-024-05548-x

**Published:** 2024-03-07

**Authors:** Alexander Tsoi, Alvaro Gomez, Carina Boström, Denise Pezzella, Jun Weng Chow, Charlotte Girard-Guyonvarc’h, Tanja Stamm, Laurent Arnaud, Ioannis Parodis

**Affiliations:** 1 Division of Rheumatology, Department of Medicine Solna, Karolinska Institutet, SE-171 76 Stockholm, Sweden; 2 Department of Gastroenterology, Dermatology and Rheumatology, Karolinska University Hospital, Stockholm, Sweden; 3 Division of Physiotherapy, Department of Neurobiology, Care Sciences and Society, Karolinska Institutet, Stockholm, Sweden; 4 Department of Occupational Therapy and Physiotherapy, Karolinska University Hospital, Stockholm, Sweden; 5 Division of Rheumatology, Department of Medicine, University Hospital of Geneva and Faculty of Medicine, University of Geneva, Geneva, Switzerland; 6 Section for Outcomes Research, Center for Medical Statistics, Informatics and Intelligent Systems, Medical University of Vienna, Vienna, Austria; 7 Ludwig Boltzmann Institute for Arthritis and Rehabilitation, Vienna, Austria; 8 Department of Rheumatology, Hôpitaux Universitaires de Strasbourg, Centre National de Référence (RESO), INSERM UMR-S 1109, Strasbourg, France; 9 Department of Rheumatology, Faculty of Medicine and Health, Örebro University, Örebro, Sweden

**Keywords:** Systemic lupus erythematosus, Healthy lifestyle, Mental health, Exercise, Diet therapy, Systematic review

## Abstract

We performed a systematic review to explore existing evidence regarding the efficacy of lifestyle interventions for the management of systemic lupus erythematosus (SLE). The search was conducted on the 22nd of June 2021 for publications between 1st of January 2000 and the date of search. Additional articles within the aforementioned timeframe and until December 2023 were added by hand searching. Databases utilized were Medline, Embase, Web of Science, and Cinahl. Lifestyle interventions were defined as any intervention encompassing one or more of the following: physical exercise, diet and nutrition, mental health, harmful exposures, sleep, and social relations. The Joanna Briggs Institute critical appraisal tools were used for risk of bias assessment. The search yielded 11,274 unique records, we assessed the full text of 199 records, and finally included 102 studies. Overall, the quality of the evidence is limited, and there were multiple sources of heterogeneity. The two domains most extensively researched were mental health (40 records) and physical exercise (39 records). Psychological interventions had a positive effect on depressive symptoms, anxiety, and health-related quality of life (HRQoL), whereas physical exercise improved fatigue, depressive symptoms, aerobic capacity, and physical functioning. Studies on diet and nutrition (15 records) support that low fat intake and Mediterranean diet may be beneficial for reducing cardiovascular risk, but large interventional studies are lacking. Studies on harmful exposures (7 records) support photoprotection and use of sunscreen. While studies imply benefits regarding disease burden and drug efficacy in non-smokers and regarding HRQoL in normal-weight patients, more survey is needed on tobacco smoking and alcohol consumption, as well as weight control strategies. Studies on social relations (1 record) and sleep (no records) were sparse or non-existent. In conclusion, psychosocial interventions are viable for managing depressive symptoms, and exercise appears essential for reducing fatigue and improving aerobic capacity and physical function. Photoprotection should be recommended to all patients. Lifestyle interventions should be considered a complement, not a substitute, to pharmacotherapy.

**Supplementary Information:**

The online version contains supplementary material available at 10.1007/s00296-024-05548-x.

## Introduction

Systemic lupus erythematosus (SLE) is a chronic autoimmune disease that primarily affects women, often in the time between puberty and menopause [1]. Typical manifestations of the disease include fatigue, rashes, and arthritis, and up to 40% of patients develop renal involvement [2]. Over time, inflammation-induced organ damage and side effects from immunosuppressive therapies lead to increased morbidity and mortality [3]. During the past decades, much focus has been placed on clinical trials for pharmaceuticals. Despite considerable advances, the response rate to new biopharmaceuticals remains limited, and substantial proportions of patients still develop organ damage and experience severe pain, fatigue, and impaired quality of life [4–7]. The use of lifestyle changes as complementary interventions has not been thoroughly studied.

The field of lifestyle medicine is rather novel. Although the definition of a lifestyle intervention is not entirely clear, the American College of Lifestyle Medicine (ACLM) and the British Society of Lifestyle Medicine (BSLM) have similar approaches when outlining what constitutes a lifestyle intervention, dividing lifestyle into six areas. ACLM lists six fundamental domains: nutrition, exercise, stress, substance abuse, sleep, and relations [8]. BSLM also specifies six domains, as follows: healthy eating, physical activity, mental well-being, minimizing harmful substances, sleep, and healthy relations [9]. Hence, these six domains i.e., physical activity and exercise, diet and nutrition, mental health, harmful exposures, sleep, and social relations, are largely common for both taxonomies.

The utilization of non-pharmacological interventions is gaining interest within the European rheumatology community. In recent years, the European Alliance of Associations for Rheumatology (EULAR) has released reviews and recommendations regarding lifestyle behaviors in people with rheumatic and musculoskeletal diseases, focusing on exercise, diet, weight, alcohol, smoking, and work participation [10–12]. A previous systematic review exploring the efficacy of lifestyle habits in patients with SLE focused on physical exercise, tobacco smoking, and diet [13], and another focused on physical activity alone [14]. There is some evidence suggesting that lifestyle has an impact on developing SLE [15].

More recently, EULAR recommendations for the non-pharmacological management of SLE and systemic sclerosis were published [16], based on a large preceding systematic literature review [17]. Although comprehensive, this review did not explicitly separate lifestyle interventions from other modalities. Hence, specific information regarding lifestyle interventions targeting modifiable elements of health experience was diluted among the multitude of explored non-pharmacological management strategies. With the present review, driven by a distinct definition of a lifestyle intervention as described above, we aimed to distill the evidence concerning six specific modifiable lifestyle facets.

## Methods

### Definition of lifestyle intervention

For the purpose of this review, we defined a lifestyle intervention as any intervention that pertains to one or more of the following six lifestyle factors: physical activity and exercise, diet and nutrition, mental health, harmful exposures, sleep, and social relations, based on the components of lifestyle medicine described by ACLM and BSLM [8, 9]. For data synthesis, we grouped the included studies into these six categories.

### Information sources and search strategy

**Fig. 1:**
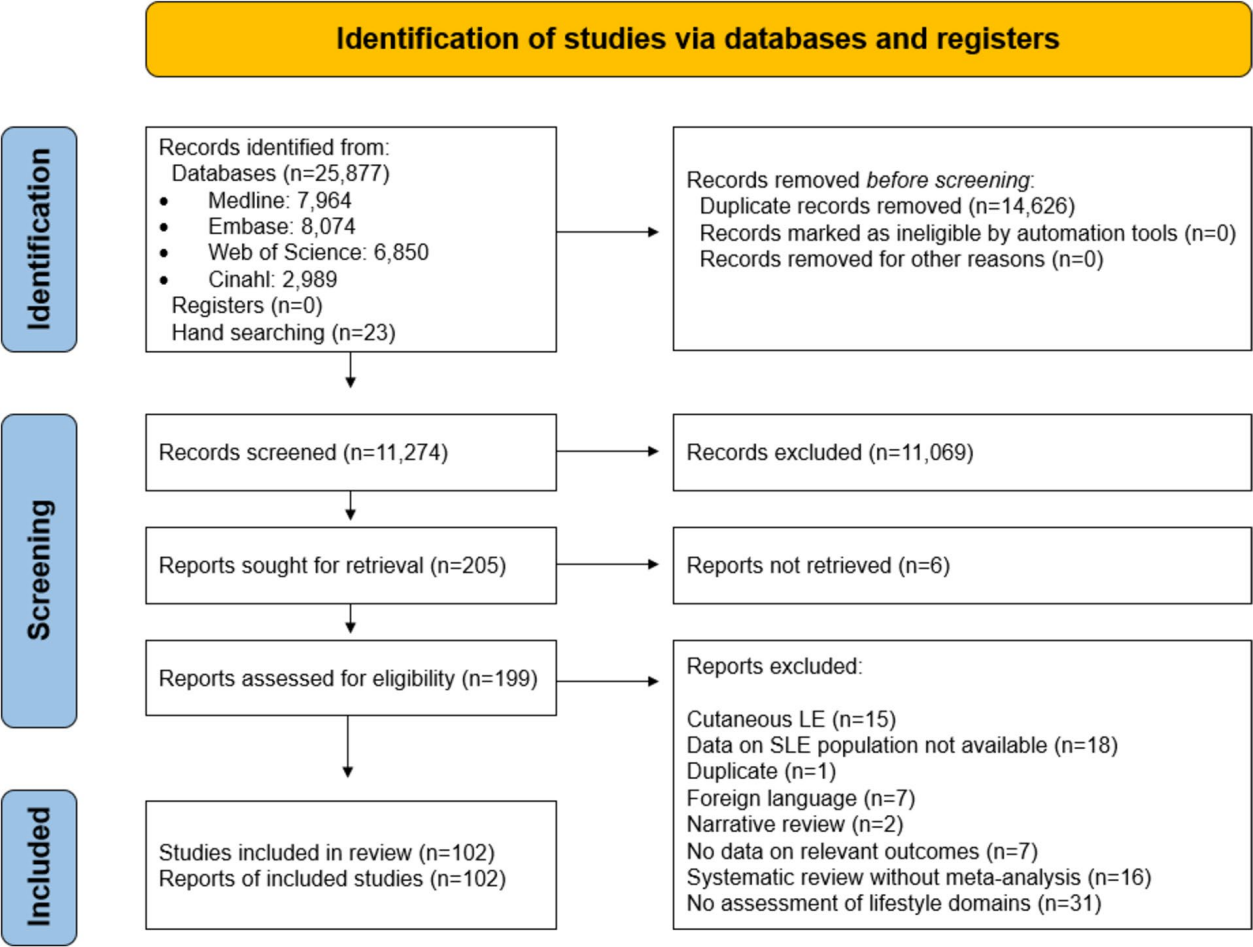
Flowchart of study selection

A systematic literature search was conducted on the 22nd of June 2021. Databases utilized were Medline (Ovid), Embase, Web of Science Core Collection, and CINAHL (EBSCO). We designed a broad search with two blocks, including SLE and an extensive list of non-pharmacological interventions, as a part of a larger systematic literature search [17], conducted to inform the EULAR recommendations for the non-pharmacological management of SLE and systemic sclerosis [16]. Two authors (AG, JWC) independently screened titles and abstracts of 11,251 potentially relevant records, under supervision of one senior investigator (IP). Additional articles within the aforementioned timeframe and until December 2023 were added by hand searching. The search and selection of studies were documented according to the preferred reporting items for systematic reviews and meta-analyses (PRISMA) statement [18], as presented in Fig. 1.

### Study selection

We included full reports that examined the efficacy of lifestyle interventions in patients with SLE (diagnosed according to classification criteria and/or ICD codes). Cross-sectional studies, case–control studies, cohort studies (with more than five participants), randomized control trials, and systematic reviews with meta-analyses were deemed eligible. We also included studies of mixed disease populations from which subgroup data on participants with SLE were extractable. We did not set restrictions for the duration of the interventions, or the time of outcome evaluation. We excluded records published in languages other than English, Spanish, or Swedish.

Due to the diverse nature of the design of the studies included, we did not set restrictions for the comparator group. Thus, studies were eligible if they compared groups receiving the lifestyle intervention of interest with standard of care, the lifestyle intervention with another intervention, or if they performed intra-individual comparisons i.e., before and after the intervention.

To be eligible, studies had to report data on efficacy for at least one of the following outcomes: disease activity, organ damage, health-related quality of life, functional impairment, pain, fatigue, depression and anxiety, psychological stress, or inflammatory markers.

Two authors (AG, JWC) independently screened the full text of the 176 records retrieved for eligibility and selected the records to be included, under supervision of one senior investigator (IP). Disagreements between reviewers were solved through consensus, together with two senior investigators (IP and CB).

### Data collection and synthesis

Two researchers (AG and DP) independently extracted information from full texts using electronic forms, including author and year of publication, study design, size of study population, intervention and/or management strategy, comparator group, outcome measures, and key findings. These data are provided in Supplementary Table S1. Considering that we were inclusive regarding study designs and outcomes, results were summarized as reported by the authors, and comprised dichotomous and continuous data, as well as effect measures for dichotomous outcomes (risk ratios and odds ratios) and continuous outcomes (mean and standardized mean differences).

### Risk of bias assessment

Risk of bias (RoB) for all included articles was conducted by one researcher (AT) using the critical appraisal (CA) checklists by the Joanna Briggs Institute [19]. Since all articles were already included before quality assessment for this review, the alternatives for overall appraisal “include”, “exclude”, and “seek further info” were modified to “robust”, “weak, and “intermediate”, respectively. The appropriate checklist for each study was selected based on study design. A study was deemed weak if there were six or more checklist items it did not clearly fulfill, intermediate if there were three to five checklist items it did not clearly fulfill, or robust if clearly fulfilling all checklist items but two or fewer. Upon RoB assessment, studies were graded by level of evidence (LoE) according to the Oxford Centre for Evidence-Based Medicine by one researcher (AT) [20].

## Results

### Study characteristics

After deduplication, the search yielded 11,274 unique records. We assessed the full text of 199 potentially relevant records, and finally included 102 studies. Of these, forty were categorized as studies on mental health [21–60], and thirty-nine as studies on physical activity and exercise [61–99]. Fifteen articles studied the effects of diet and nutrition [100–114], whereas seven studies examined the reduction of harmful exposures [115–121]. One study evaluated the effects of social relations [122], and no studies were found evaluating the effects of sleep.

### Mental health

Out of forty studies examining the effects of mental health management, many focused on cognitive–behavioral therapy (CBT) [24, 26, 30–32, 37, 43, 48]. Two studies were meta-analyses, combining studies utilizing different psychological interventions [35, 38]. One meta-analysis found that interventions used in the constituent studies had an effect on anxiety, depressive symptoms, stress, and disease activity [35]. The other found that psychological interventions could improve depressive symptoms, and certain aspects of one’s health-related quality of life (HRQoL), but not disease activity [38]. Neither meta-analysis found any effect on fatigue [35, 38]. Three studies where randomized controlled trials (RCT) graded as LoE 2. One of these RCTs evaluated disease activity, and did not find that the intervention (biofeedback-assisted CBT) had any effect [24]. The other two RCTs showed that counseling may have a positive effect on anxiety [59] and sexual function [60], respectively. Table 1 shows the included studies of LoE 1 and 2.

**Table 1:**
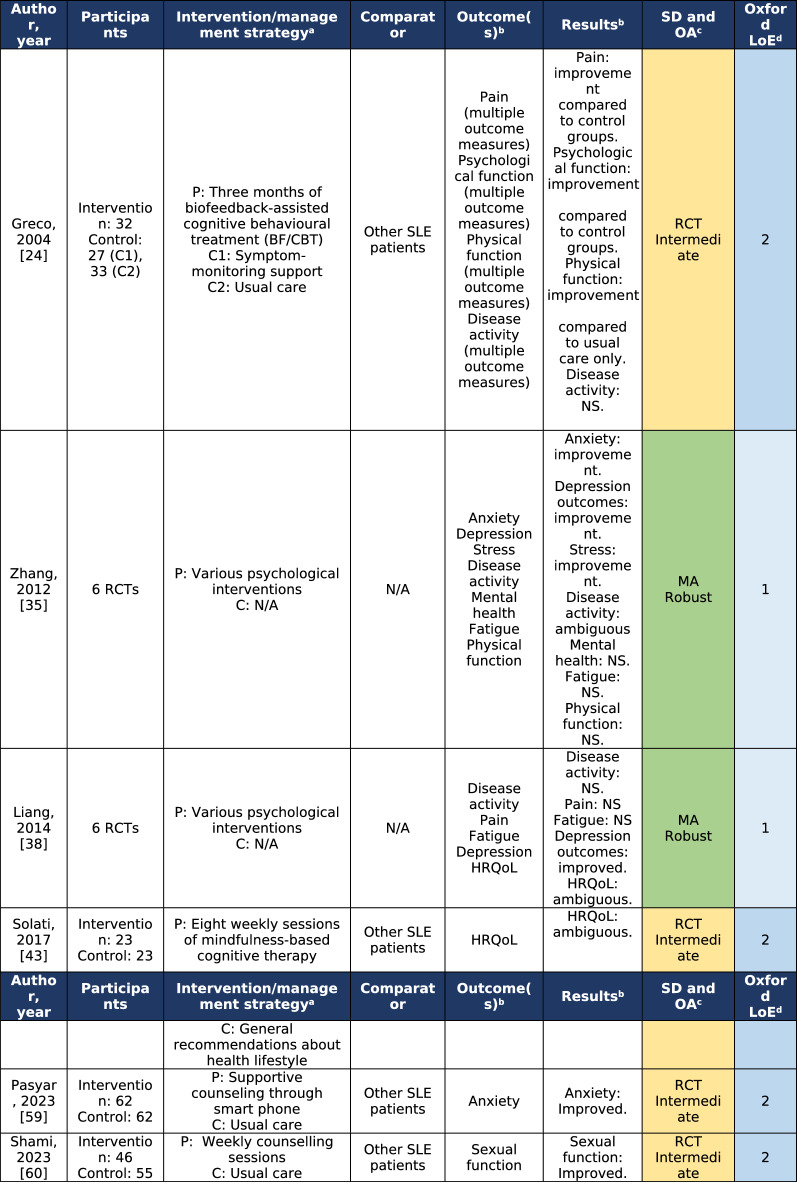
Studies on mental health management for systemic lupus erythematosus [24, 35, 38, 43, 59, 60]

^a^*P* intervention/management strategy applied to population under investigation, *C* intervention/management strategy applied to participants in the comparator group, *N/A* not applicable

^b^*HRQoL* health-related quality of life, *NS* non-significant

^c^Study design and overall appraisal (adapted from the Joanna Briggs Institute Manual for Evidence Synthesis [19]); *green colour* denotes a robust study and *yellow colour* denotes intermediate robustness in overall appraisal; *MA* meta-analysis, *RCT* randomized controlled trial

^d^*LoE* levels of evidence according to the Oxford Centre for Evidence-Based Medicine [20]; *light blue colour* denotes level 1 (strongest evidence) and *dark blue colour* denotes level 2

### Physical activity and exercise

Of the thirty-nine studies that investigated effects of physical activity and exercise, four were meta-analyses [80, 81, 86, 94]. Results showed that exercise in patients with SLE could improve HRQoL [94], decrease fatigue [80, 81], and decrease depressive symptoms [80], but not ameliorate disease activity [80]. The individual RCTs assessed as intermediate or robust in CA also found that exercise could improve fatigue [62] and depressive symptoms [76], but had no effect on disease activity [62, 76, 78] or prevention of organ damage [78]. Studies with level of evidence 1 and 2 are presented in Table 2.

**Table 2:**
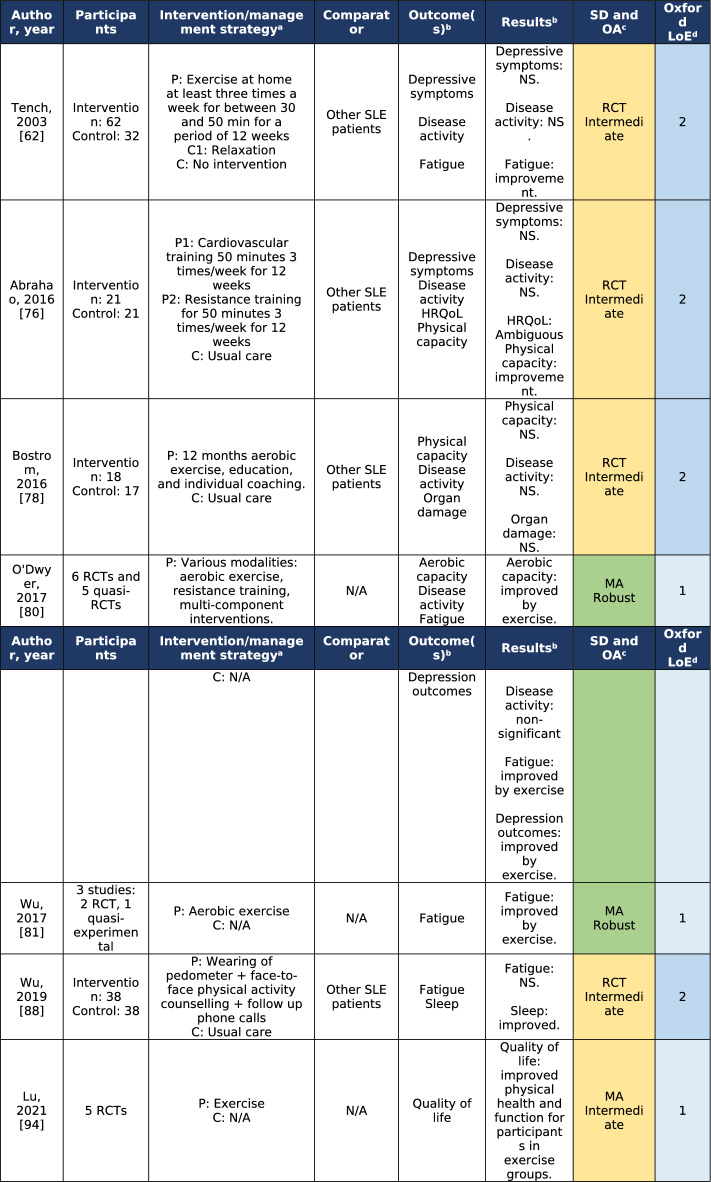
Studies on physical activity and exercise for the management of systemic lupus erythematosus [62, 76, 78, 80, 81, 88, 94]

^a^*P* intervention/management strategy applied to population under investigation, *C* intervention/management strategy applied to participants in the comparator group

^b^*HRQoL* health-related quality of life, *NS* non-significant

^c^Study design and overall appraisal (adapted from the Joanna Briggs Institute Manual for Evidence Synthesis [19]); *green colour* denotes a robust study and *yellow colour* denotes intermediate robustness in overall appraisal; *MA* meta-analysis, *RCT* randomized controlled trial

^d^*LoE* levels of evidence according to the Oxford Centre for Evidence-Based Medicine [20]; *light blue colour* denotes level 1 (strongest evidence) and *dark blue colour* denotes level 2

The notion that physical exercise does not affect disease activity is further consolidated in other studies of non-RCT design [70, 71].

### Diet and nutrition

Of the fifteen studies that investigated diet and nutrition, one was a systematic review with meta-analyses rated as intermediate in critical appraisal, whose main finding regarding SLE was that fish oil or omega-3 extracts did not affect disease activity. Two were RCTs rated as intermediate in critical appraisal. One of these suggested that turmeric extract may decrease proteinuria, hematuria, systolic blood pressure, and complement consumption [108]. However, this trial lacked between-groups comparisons in statistical analysis, so whether turmeric outperformed placebo was unclear. The other of these trials found that daily intake of green tea extract could lower disease activity after a 12-week regimen [110].

Studies with a larger sample size of non-RCT design (LoE 3; CA: robust) showed associations between (i) high intake of vitamin C [101], (ii) vitamin B6, and (iii) total dietary fiber [105] and lower disease activity, and between high vegetable fat intake and increased cardiovascular risk [101]. A cross-sectional study on the Mediterranean diet found this to be associated with both lower levels of disease activity and less organ damage accrued [112]. Table 3 shows the included studies on diet and nutrition of LoE 2 (no LoE 1 articles were found).

**Table 3:**
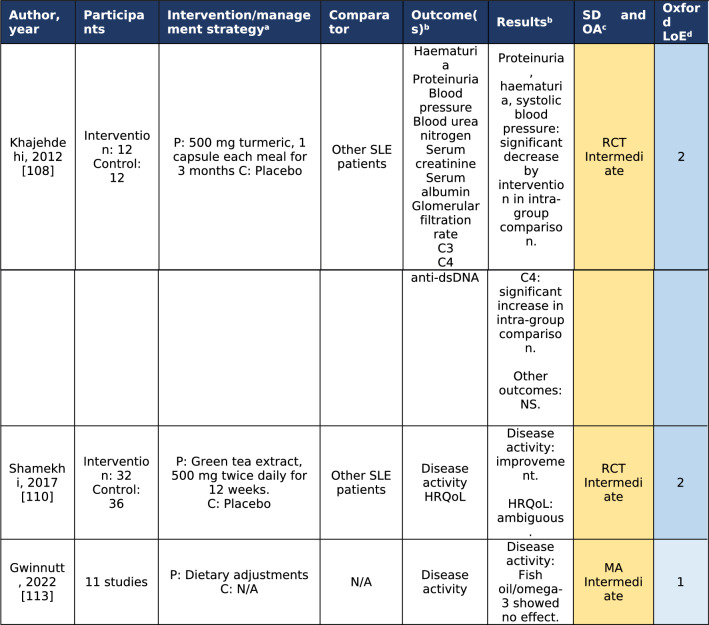
Studies on diet and nutrition for the management of systemic lupus erythematosus [108, 110, 113]

^a^*P* intervention/management strategy applied to population under investigation, *C* intervention/management strategy applied to participants in the comparator group, *N/A* not applicable

^b^*HRQoL* health-related quality of life, *NS* Non-significant

^c^Study design and overall appraisal (adapted from the Joanna Briggs Institute Manual for Evidence Synthesis [19]); *yellow colour* denotes intermediate robustness in overall appraisal; *MA* meta-analysis, *RCT* randomized controlled trial

^d^*LoE* levels of evidence according to the Oxford Centre for Evidence-Based Medicine [20]; *light blue colour* denotes level 1 (strongest evidence) and *dark blue colour* denotes level 2

### Harmful exposures

Of seven studies examining the relationship between SLE and harmful exposures, four examined photoprotection [115–117, 120]. Sunscreen has been shown to reduce expression of inflammatory markers in sun-exposed skin [117] as well as UV-radiation-induced skin lesions [115]. However, photoprotection awareness seems to have no effect on disease activity, organ damage, or lupus serology [120]. Of the other three studies, two focused on smoking [119, 121] and the other on exposure to household products [118]. Smoking was found to be associated with higher disease activity [119] and increased serum levels of B cell activating factor (BAFF) [121], but not with changes in antibody profile [119]. Use of bath salts was associated with a higher number of self-reported flare days, whereas other household products, such as adhesives and paint, were associated with less self-reported flare days, with relative risks from household product exposures ranging between 0.99 and 1.01 [118].

### Social relations

One study was found evaluating social relations; this was cross-sectional in nature and found a negative association between illness uncertainty and support availability in hospitalized lupus patients [122]. From this, the authors concluded that social support should be made more accessible to these patients.

### Sleep

None of the included studies examined the impact of sleeping habits.

## Discussion

This systematic review examined published literature from over two decades across multiple electronic databases, aiming to summarize the evidence of lifestyle interventions for the management of systemic lupus erythematosus, a concept which has hitherto been loosely defined. Based on the emerging field of lifestyle medicine, we defined a six-domain construct to classify interventions pertaining to lifestyle. Of these six domains, mental health and physical activity and exercise were the most studied, whereas the impact of social relations and sleep appeared under-explored. Cognitive behavioral therapy seemed to reduce depressive symptoms, whereas aerobic exercise emerged as an efficacious lifestyle intervention for the amelioration of fatigue and depressive symptoms, and for the improvement of aerobic capacity.

Studies on mental health management, among which CBT was the most studied modality, showed efficacy of psychosocial interventions for improving anxiety and depressive symptoms, as well as certain aspects of HRQoL. The notion of psychological interventions reducing disease activity as reported in one meta-analysis of RCTs, although highly appealing, was not supported by the individual constituent studies used in the meta-analysis, all of them also separately included in this review, which did not show any clear effect on disease activity [24, 25, 30, 123]. Hence, this finding should be interpreted with caution.

Physical activity has a large body of evidence backing several health benefits in the general population, and a recent systematic review consolidated that aerobic training and resistance training yielded improvements in cardiovascular risk and physical function, respectively [14]. This complements the findings that physical exercise can be efficacious in managing fatigue and depressive symptoms.

Studies on diet and nutrition could be categorized into two groups: studies assessing dietary supplements, and studies assessing specific diets. The dietary supplements evaluated were broad, and included turmeric acid, green tea, and micronutrient supplements. Although some of those showed an effect on disease activity, studies included small sample sizes, had short follow-up periods, and were observational or reliant on self-reported data, precluding definite conclusions. Concerning diets, low glycemic [106], the Mediterranean [112], and the NCEP Step 2 [100] diets were explored. Among them, a study by Pocovi-Gerardino et al. assessing Mediterranean diet found improvements in body composition, cardiovascular risk, disease activity and organ damage [112]. While these findings are promising, they rely on food questionnaire data, and interventional studies evaluating the efficacy of Mediterranean diet are awaited to draw more definite conclusions.

Exposure to different elements are well-known to affect the course of SLE; naturally, sunscreen that has been shown to be efficacious [115, 117] can be complemented with other forms of photoprotection, such as wearing a hat or staying in the shade. Besides being associated with higher disease activity, smoking has been shown to reduce the efficacy of pharmacological treatment, specifically antimalarial agents [124] and belimumab [125, 126]. Another factor that is not touched upon in any of the included studies is cold exposure, which if mitigated can assist the management of Raynaud’s phenomenon [16]. Despite a sparsity of data in literature, protection from cold is now included in the EULAR recommendations for the non-pharmacological management of SLE [16]. Lastly, overweight and obesity have been associated with poor HRQoL, warranting investigation of weight loss strategies [127–129].

This review clarifies which lifestyle interventions have proven efficacious in relation to which outcomes, aspiring to serve as a guidance for clinicians toward implementation of such strategies. Our review also reveals the scarcity of high-level evidence regarding such interventions, and thus the need for further research, aiding researchers in the field form their research agenda.

A major drawback was that the identified studies frequently provided a poor level of evidence; there was a dearth of meta-analyses and systematic reviews that incorporated high-quality RCTs. Another concern was the substantial degree of heterogeneity across studies. This heterogeneity encompassed variations in the approach and length of interventions, evaluation instruments, comparison groups, and areas of outcomes. Nevertheless, keeping those diverse served the purpose of the broad and inclusive research questions. Some studies examined two or more interventions in parallel [66, 78], making it hard to evaluate the effect of each separate modality. Moreover, some lifestyle factors remain under-researched, and no firm conclusions can be drawn for those. Lastly, although we aligned with the lifestyle intervention categories suggested by lifestyle medicine organizations in the US and the UK, it is likely that some relevant interventions were not captured. Importantly, the identified drawbacks limit the generalizability of the results to all people living with SLE.

In conclusion, psychological interventions for mental health management and physical activity and exercise were the most studied interventions. While psychological interventions have a positive effect on depressive symptoms, anxiety, and quality of life, physical exercise improves fatigue, depressive symptoms, and physical functioning. Low-fat and Mediterranean diets constitute appealing strategies for reducing the cardiovascular risk and enhance disease control, but larger interventional studies are still required. Photoprotection remains an evidence-based recommendation for avoidance and management of cutaneous involvement. Insights regarding social relations and sleep are lacking, wherefore future studies may aim to fill this gap in knowledge. Importantly, there is an overall stronger body of evidence for medicinals for SLE [130, 131], and although several studies included in this review support efficacy of lifestyle interventions in patient-reported outcomes, hardly any evidence is provided for lifestyle interventions modulating disease activity. Hence, lifestyle interventions should be considered a complement, not a substitute, to pharmacotherapy in SLE.

### Supplementary Information

Below is the link to the electronic supplementary material.

Supplementary file1 (PDF 546 KB)

## Data Availability

Full data relating to this review can be made available by the corresponding author upon reseanable request.
