# Supplementary material for: Efficacy of lifestyle interventions in the management of systemic lupus erythematosus: a systematic review of the literature
Source: Rheumatol Int. 2024 Mar 7;44(5):765–78. doi: 10.1007/s00296-024-05548-x (PMC10980639; doi:10.1007/s00296-024-05548-x)
Supplement: Supplementary file 1 — Supplementary file1 (PDF 546 KB) [file 296_2024_5548_MOESM1_ESM.pdf]

Supplementary Table S1

Study characteristics

Author, year	Participants	Intervention/management strategy[a]	Comparator	Outcome(s)[b]	Results	SD&OA[c]	Oxford LoE[d]
**Mental health**							
Sohng, 2003 (1)	Intervention: 21<br>Control: 20	P: Attend a self-management course<br>C: Usual care	Other SLE patients	Multidimensional Assessment of Fatigue<br>BDI<br>VAS scale for pain<br>7 items devised by Arthritis Foundation<br>10 items devised by Arthritis Foundation	Fatigue<br>IG: before intervention: 27.7 (10.3)<br>after intervention: 24.8 (10.4)<br>CG: before intervention: 21.7 (9.6)<br>after intervention: 25.5 (10.1)<br>p=0.049<br><br>Depression<br>IG: before intervention:12.8 (9.1)<br>after intervention: 11.1 (9.0)<br>CG: before intervention: 8.3 (7.3)<br>after intervention: 10.9 (5.0)<br>p=0.025<br><br>Pain<br>No significant differences in the mean scores for pain<br>p=0.469<br><br>Self-efficacy<br>Self-efficacy improved after the intervention<br>p=0.001<br><br>Coping skills<br>IG: before intervention: 65.2 (19.8)<br>after intervention: 68.2 (20.3)/<br>CG: before intervention:71.7 (16.1)<br>after intervention: 68.6 (16.3)<br>p=0.007	10<br>I	3
Brown, 2004 (2)	Intervention: 5 with SSc, 5 with SLE<br>Control: N/A	P: Educational programme<br>C: Educational programme	SSc patients	Results of interview	Overall satisfaction with both programmes, however SLE revealed a more positive feeling about their attendance. Both groups felt that it was valuable to meet individuals with the same disease and welcomed an educator within the program planning team. Both groups were unanimously satisfied with the content and format. Behaviour-wise, SLE patients revealed more definite life changes than SSc patients.	9<br>R	4
Dorsey, 2004 (3)	Intervention: 34<br>Control: 71	P: Attend support group<br>C: No	Other SLE patients	SF-36 (PCS and MCS)<br>SLAM	HRQoL<br>Adjusted MCS<br>Support group: 31.5 (1.9)<br>No support group: 39.8 (1.3)<br>p<0.05<br><br>Adjusted PCS<br>Support group: 38.0 (1.6)<br>No support group: 39.8 (1.1)<br>p>0.05	1<br>R	3

Author, year	Participants	Intervention/management strategy[a]	Comparator	Outcome(s)[b]	Results	SD&OA[c]	Oxford LoE[d]
Greco, 2004 (4)	Intervention: 32<br>Control: 27+33	P: Receive biofeedback-assisted cognitive behavioural treatment (BF/CBT)<br>C: Usual care	Other SLE patients	AIMS2-Pain<br>MPI-I<br>CES-D<br>Arthritis Self-Efficacy Scale<br>PSS (Perceived Stress Scale)<br>SF-36<br>SLAM-R<br>SLEDAI	Pain<br>Pain and psychological dysfunction= BF/CBT had significantly greater reductions compared with SMS group (p=0.044) and UC group p=0.028<br><br>Psychological functioning<br>BF/CBT greater reductions compared with SMS group (p<0.001) and UC group p<0.001)<br>9-month follow up<br>BF/CBT continued to exhibit relative benefit compared with UC in psychological functioning (p=0.023)<br>Physical functioning<br>BF/CBT significantly greater improvement compared with UC(p=0.035), and marginally significant improvement relative to SMS (p=0.097)<br><br>Disease activity<br>changes in BF/CBT group not significantly different from those found for the SMS group (p=0.220) or the UC group (p=0.372)	11<br>I	2
Karlson, 2004 (5)	Intervention: 64<br>Control: 58	P: Discussion between educator, patient, and partner, after a regular visit for medical care + telephone follow up<br>C: 45-minute video presentation about lupus, and monthly telephone calls	Other SLE patients	SF-36<br>SLAQ<br>Profile of mood states (POMS)<br>Self-efficacy–scale<br>modified social support scale developed for arthritis patients<br>8-item subscale of the Medical Interview Satisfaction Scale	HRQoL<br>SF-36 MCS:<br>Intervention: 69 (26).<br>Control: 58 (23)<br>p=0.04.<br><br>SF-36 PCS:<br>Intervention: 55 (25).<br>Control: 48 (25)<br>p=0.20.<br><br>Disease activity<br>No significant differences in SLAQ scores.<br>Fatigue<br>Intervention: 5.1 (2.4).<br>Control: 6.3 (2.4)<br>p=0.02.<br>Self-efficacy<br>Intervention: 7.2 (1.9).<br>Control: 6.2 (2.0)<br>p=0.02.<br>Social support<br>Intervention: 4.4 (0.6).<br>Control: 4.1 (0.6)<br>p=0.03.	11<br>W	3
Goodman, 2005 (6)	Intervention: 11<br>Control: 22	P: Application of the Cognitive-Behaviour Therapy based on the Chronic Illness Self-Management Course<br>C: Usual care	Other SLE patients	SF-36<br>HADS<br>CES-D (Centre for epidemiologic al Studies-Depression scale)<br>CDS (Cognitive Distortion Scale)<br>Fatigue Intensity scale	Physical health status<br>No significant differences in SF-36 PCS scores.<br><br>Anxiety and depression<br>Significant reduction in total HADS score (p<0.01), but not in CES-D depression.<br><br>Dysfunctional cognitions<br>Significant reductions in self-criticism (p<0.002) and	10<br>I	3

Author, year	Participants	Intervention/management strategy[a]	Comparator	Outcome(s)[b]	Results	SD&OA[c]	Oxford LoE[d]
				Illness Perceptions Questionnaire revised (IPQ-R)<br>Perceived Stress Scale (PSS)	helplessness (p<0.01), but not in other domains.<br><br>Fatigue<br>Significant main effect, favouring the intervention group (p<0.02)<br><br>Illness representations<br>Significant main effect, favouring the intervention group in identity, treatment control and emotional representations (p<0.05).<br><br>Stress<br>Significant main effect, favouring the intervention group (p<0.02)		
Harrison, 2005 (7)	Intervention: 17<br>Control: -	P: Attend a psychoeducational group combining functional strategy training and psychosocial support (MINDFULL program)<br>C: N/A: intraindividual assessment	N/A: intraindividual assessment	BDI<br>Metamemory in Adulthood Questionnaire (MIA)<br>Memory Functioning Questionnaire (MFQ)<br>SDI	Depression<br>BDI: improved from 16.3 (9.5) to 10.9 (5.0)<br>p=0.022<br><br>Metamemory<br>before MINDFULL: 3.42 (0.14)<br>after MINDFULL: 3.65 (0.20)<br>p=0.00003<br><br>Memory Functioning<br>Kinds of memory problems 2.88 (0.96) 3.94 (0.85) p=0.003<br>Frequency of forgetting 4.16 (0.58) 4.47 (0.81) p=0.123<br>Seriousness of forgetting 2.98 (0.68) 3.10 (0.85) p=0.649<br>Mnemonic usage 2.30 (0.88) 1.84 (0.59) p=0.032<br>Retrospective functioning 2.29 (0.97) 3.08 (1.29) p=0.020	10<br>I	4
Haupt, 2005 (8)	Intervention: 26<br>Control: 8	P: Group session focused on psychoeducative and psychotherapeutic elements<br>C: Same intervention 6 months later (waiting group)	Other SLE patients	SCL-90-R (Symptom Checklist 90-Revised)<br>SF-36<br>HADS-D<br>KKG (control convictions relating to illness and health)<br>Freiburg questionnaire on coping with illness (FKV)<br>Self-acceptance registration scale (SESA)<br>Everyday life questionnaire (FAL)	Psychological distress<br>Aggression domain: 51.62 (10.20) p<0.001<br><br>Quality of life<br>Social functioning domain: 67.65 (27.20) p<0.05<br><br>Depression<br>HADS-D 6 months: 5.38 (3.67) p<0.01<br><br>Control convictions<br>Follow up (12 months) 40.26 (33.74) p<0.05<br><br>Social life<br>Follow up (12 months) 35.15 (5.71) p<0.01	10<br>I	3
Miljeteig, 2009 (9)	Intervention: 13<br>Control: -	P: Take part in a patient education program<br>C: N/A: intraindividual assessment	N/A: intraindividual assessment	SF-36<br>VAS for Pain<br>VAS for fatigue<br>VAS for physical well-being	Quality of life<br>General health= from 47.3 (17.5) to 56.0 (13.2)<br>p=0.029<br>Mental health= from 76.7 (SD 16.3) to 84.0 (10.9)<br>p=0.091	9<br>R	4

Author, year	Participants	Intervention/management strategy[a]	Comparator	Outcome(s)[b]	Results	SD&OA[c]	Oxford LoE[d]
				Patient satisfaction questionnaire	Pain<br>Non-significant (no detailed data provided)<br><br>Fatigue<br>Non-significant (no detailed data provided)<br><br>Physical well-being<br>Non-significant (no detailed data provided)		
Navarrete-Navarrete, 2010 (10)	Intervention: 21<br>Control: 24	P: Attend 10 Cognitive-Behaviour Therapy sessions<br>C: General recommendations about health lifestyle	Other SLE patients	SF-36<br>SLEDAI<br>Stress Vulnerability Inventory<br>Perception of stress (SRLE)<br>BDI<br>STAI-T	Quality of life<br>Physical role:<br>T0–T3= TG: 0.15<br>CG: 0.30<br>T0–T9= TG: 0.20<br>CG: 0.45<br>T0–T15= TG: 0.40<br>CG: 0.47<br><br>Disease activity<br>No significant changes<br>T3 p=0.085<br>T9: p=0.268<br>T15: p=0.688<br><br>Stress<br>T3<br>TG: 7.8 (4) p=0.017<br>CG: 11.6 (6)<br>T9<br>TG: 7.5 (6.6) p=0.050<br>CG: 11.3 (6.1)<br>T15<br>TG: 6.3 (6.3) p=0.001<br>CG: 12.1 (5.5)<br><br>Depression<br>T3<br>TG: 7.8(6.6) p=0.006<br>CG: 16.6 (11.2)<br>T9<br>TG :10.3 (9.4) p=0.161<br>CG: 17.1 (13.1)<br>T15<br>TG: 7.6(7.2) p=0.003<br>CG: 16.5 (10.8)<br><br>Anxiety<br>T3<br>TG 44 (31) p=0.008<br>CG: 69.1 (26.3)<br>T9<br>TG: 43.4 (33.6) p=0.064<br>CG: 62.2 (30.4)<br>T15<br>TG: 42.4 (26.4) p=0.007<br>CG: 66.5 (27.3)	11<br>W	3
Navarrete-Navarrete, 2010 (11)	Intervention: 18<br>Control: 16	P: Attend 10 Cognitive-Behaviour Therapy sessions<br>C: General recommendations about health lifestyle	Other SLE patients	Cohen Perceived Stress Questionnaire<br>Perceived Stress Scale (SRLE)<br>SF-36<br>State–Trait Anxiety	Quality of life<br>MCS: [F= (1.19).0.627 p<0.035]<br>PCS: [F= (1.19).0.434 p<0.078]<br><br>Anxiety (predictor of MCS)<br>R2 corrected: 0.689, T: - 7.294, p<0.00	11<br>W	3

Author, year	Participants	Intervention/management strategy[a]	Comparator	Outcome(s)[b]	Results	SD&OA[c]	Oxford LoE[d]
				Inventory (STAI)<br>Beck Depression Inventory (BDI)			
Brown, 2012 (12)	Intervention: 27<br>Control: 10 +16	P: Follow three separate CBT modules preinstalled on a CD ROM<br>C: Educational sessions usual care	Other SLE patients	McGill Pain Questionnaire – Short Form (SF-MPQ)<br>Behaviour Assessment System for Children (BASC)<br>Self-Perception Profile for Adolescents (SPPA)<br>Multidimensional Health Locus of Control Scales (MHLC)<br>PedsQL<br>SLEDAI<br>Perceived Social Support-Family (PSS-Fa)<br>Perceived Social Support-Friend (PSS-Fr)<br>The Coping Strategies Questionnaire (CSQ)	Pain<br>No significant differences<br><br>Behaviour<br>No significant differences<br><br>Self-perception<br>No significant differences<br><br>Self-efficacy<br>No significant differences<br><br>Quality of life<br>No significant differences<br><br>Disease activity<br>No significant differences<br><br>Social support<br>No significant differences<br><br>Coping skills<br>Increase at post-hoc secondary analysis<br>($p<0.05$)	11<br>W	3
Drenkard, 2012 (13)	Intervention: 45<br>Control: -	P: Attend the Chronic Disease Self-Management Program (CDSMP)<br>C: N/A: intraindividual assessment	N/A: intraindividual assessment	SF-36<br>Self-efficacy for Managing Chronic Disease Scale<br>Cognitive Symptom Management Scale<br>Exercise Behavior Scale<br>three-item Communication with Physicians Scale<br>Self-reported Medication-taking Scale<br>Grady Health Electronic Records.	Quality of life<br>Mean change=2.4, $p=0.032$<br><br>Self-efficacy<br>Mean change=0.5, $p=0.035$<br><br>Self-management behaviours<br>Mean change=0.3, $p=0.036$	10<br>I	4
Ganachari, 2012 (14)	Intervention: 21<br>Control: 20	P: Education regarding SLE and its management including lifestyle modifications<br>C: Usual care	Other SLE patients	Modified Morisky Scale (MMS)<br>Knowledge assessment questionnaire	Medication adherence<br>Test group: pre-counselling: 3<br>post-counselling: 5.8<br>Control group: pre-counselling: 2.9<br>post-counselling: 4.6<br>$p<0.05$	11<br>W	3

Author, year	Participants	Intervention/management strategy[a]	Comparator	Outcome(s)[b]	Results	SD&OA[c]	Oxford LoE[d]
					Medication knowledge<br>Test group: m0: 5.52<br>m2: 16.13<br>Control group: m0 6.68<br>m2: 7.54<br>p<0.001		
Zhang, 2012 (15)	6 RCTs	P: Cognitive-Behaviour Therapy sessions, supportive therapy, multiple psychological interventions, psychoeducational intervention.<br>C: N/A	N/A	Self-rating anxiety scale (SAS)<br>HAMA<br>STAI-T<br>BDI<br>CES-D<br>Self-rating depression scale (SDS)<br>HAMD<br>SLEDAI<br>SLAM<br>SLAQ<br>FSS<br>SF-36<br>Cohen's perceived stress scale (STRESS)<br>Revised Hasstes scale	Anxiety<br>Therapy groups: significant improvement versus control (standard mean difference: = –0.95, 95% CI: (-1.57, -0.34), p<0.001).<br><br>Depression<br>Therapy groups: significant improvement versus control (standard mean difference: = –1.14, 95% CI: (-1.84, -0.44), p<0.001).<br><br>Disease activity<br>Therapy groups: significant improvement versus control (standard mean difference: = –0.34, 95% CI: (-0.57, -0.11), p<0.001).<br><br>Fatigue<br>Therapy groups: no significant difference versus control (mean difference: = –0.17, 95% CI: (-0.49, 0.15), p=0.30).<br><br>HRQoL<br>Therapy groups: significant improvement versus control in physical function (standard mean difference: = 7.65, 95% CI: (0.16, -15.13), p=0.05), but not in mental health.<br><br>Stress<br>Therapy groups: significant improvement versus control (standard mean difference: = -0.63, 95% CI: (-1.02, -0.23), p<0.001).	12<br>R	1
Bantornwan, 2014 (16)	Intervention: 15<br>Control: 15	P: Meditation instruction + meditation practice<br>C: Usual care	Other SLE patients	SF- 36<br>Normetanephrine levels<br>Heart rate variability	Sympathetic activity<br>Serum normetanephrine level decreased in meditation and control groups, not statically significant.<br><br>Heart rate:<br>Meditation: 78.5 (4.7)<br>Control: 82.2 (4.2)<br>p=0.03<br><br>Heart rate variability [measured only in intervention group]<br>Improvement in time and frequency domains.<br><br>Quality of life<br>SF-36 PCS<br>Meditation: m0: 21.4(5.0-50-2)<br>m6 62.2 (51.8-88.4)<br>Control: m0: 19.4 (10.4-49.2)<br>m6: 55.4 (36.4-83.4). p=0.04	10<br>I	3

Author, year	Participants	Intervention/management strategy[a]	Comparator	Outcome(s)[b]	Results	SD&OA[c]	Oxford LoE[d]
					SF-36 MCS<br>Meditation: m0: 16.9 (4.4-46.0)<br>m6 72.4 (45.1-81.6)<br>Control: m0: 13.9 (7.7-44.2)<br>m6: 45 (29.8-77.6). p<0.01		
Jolly, 2014 (17)	Intervention: 10<br>Control: 5	P: Follow a modified BI-CBT 8 step program + Skin care education + appearance enhancement workshop<br>C: Usual care	Other SLE patients	Body image in Lupus Scale (BILS)<br>Multidimensional Body Self-Relations Centre for Epidemiological Studies-Depression (CES-D), STAI<br>LupusPRO<br>SELENA-SLEDAI<br>SDI	Body image<br>Intervention group:<br>SIBID pre: 1.92 (0.29)<br>SIBID 24 weeks: 1.16 (1.05) p=0.048<br>BASS pre: 2.38 (0.84)<br>BASS 24 weeks: 2.96 (0.76) p=0.008<br>Control group:<br>No significant changes<br><br>Depression<br>CES-D Intervention group:<br>Pre: 22.60 (11.67)<br>24 wk: 16.00 (11.65) p=0.045<br>Control group:<br>No significant changes<br><br>Quality of Life<br>Intervention group:<br>pre: 40.00 (30.37)<br>24 wk: 79.38 (27.18) p=0.001<br>Control group:<br>No significant changes<br><br>Disease activity<br>Intervention group:<br>SLEDAI total score: pre 5.60 (4.88)<br>SLEDAI total score 24 wk: 6.25 (3.77) p=1	10<br>I	3
Liang, 2014 (18)	6 RCTs	P: Cognitive-Behaviour Therapy sessions, psychoeducational intervention, expressive group psychotherapy.<br>C: N/A	N/A	BDI<br>SLEDAI<br>FSS<br>SF-36<br>SF-36<br>AIMS2-Pain<br>VAS pain	Depression<br>Std. mean difference: -0.44 (-0.78 – --0.10). p=0.01<br><br>Disease activity<br>Std. mean difference: -0.68 (-1.82–0.46). p=0.24<br><br>Fatigue<br>Std. mean difference: 0.10 (-0.19–0.39). p=0.51<br><br>HRQoL<br>PCS<br>Std. mean difference: 8.85 (3.69–14.0). p<0.001<br><br>MCS<br>Std. mean difference: 14.4 (-4.9–33.8). p=0.14<br><br>Pain<br>Std. mean difference: 0.35 (-0.23–0.93). p=0.23	12<br>R	1
Williams, 2014 (19)	Intervention: 15<br>Control: 15	P: Follow the "Better Choice, Better Health" Chronic Disease Self-Management Program (CDSMP)<br>C: Usual care	Other SLE patients	STAI<br>DHEA and cortisol levels in saliva sample<br>Arthritis Self-Efficacy Scale pain<br>MOS<br>BDI	Pain<br>d=0.96<br><br>Psychological distress<br>PI<br>d=1.13<br>4 months:<br>d=0.78<br><br>Depression	11<br>W	3

Author, year	Participants	Intervention/management strategy[a]	Comparator	Outcome(s)[b]	Results	SD&OA[c]	Oxford LoE[d]
				LUP-QOL	PI:<br>d=1.63<br>4 months:<br>d=1.68<br><br>Social/role activities limitation<br>Difference between baseline and PI<br>LSES: −0.36<br>STAI: −0.24<br>BDI-II: 0.40 p<0.05<br>CSM: −0.38<br>HSD: 0.50 p<0.05		
Williams, 2014 (20)	Intervention: 15<br>Control: 15	P: Take part in BLESS (Balancing Lupus Experience with Stress Strategies) study<br>C: Usual care	Other SLE patients	Medical Outcomes Study (MOS) health distress scale<br>Arthritis Self-Efficacy Scale<br>BDI<br>LUP-QOL<br>State-Trait Anxiety Inventory (STAI)	Health distress<br>Mean difference Post intervention= IG: -0.94<br>CG: 0.31<br><br>Self-efficacy<br>Mean difference Post intervention= IG:19.17<br>CG: -3.21<br><br>Depression<br>Mean difference Post intervention= IG: -7.21<br>CG: 2.89<br><br>Anxiety<br>Mean difference Post intervention= IG: 0.58;<br>CG: −1.81	11<br>W	3
Horesh, 2017 (21)	Intervention: 6<br>Control: -	P: Participate in a mindfulness group protocol<br>C: N/A: intraindividual assessment	N/A: intraindividual assessment	Qualitative	Qualitative improvement	4<br>W	5
O'Riordan, 2017 (22)	Intervention: 21<br>Control: -	P: Take part in FAME (Fatigue and Activity Management Education) = 1 h group education / 1 h individual goal<br>C: N/A: intraindividual assessment	N/A: intraindividual assessment	FSS<br>FAI<br>SEPECSA<br>HADS-A<br>HADS-D<br>LupusQoL	Fatigue<br>nonsignificant improvements<br>T1/T2 p=.370<br>T2/T3 p=1.000<br>T1/T3 p=0.306<br><br>Self-Efficacy<br>nonsignificant improvements<br>T1/T2 p=0.126<br>T2/T3 p=0.4572<br>T1/T3 p=0.056<br><br>Anxiety<br>nonsignificant improvements<br>T1/T2 p=0.722<br>T2/T3 p=0.229<br>T1/T3 p=0.342<br><br>Depression<br>T1 median:6<br>T3 median:4<br>T1/T3 p=0.050<br><br>Quality of life<br>Category “burden to others”:<br>T1 median 53.17<br><br>T2 median: 63.10<br>T3 median: 55<br>T1/T2=0.046<br>T2/T3= 0.033<br>Category “fatigue”:<br>T1 median 38.99	10<br>I	4

Author, year	Participants	Intervention/management strategy[a]	Comparator	Outcome(s)[b]	Results	SD&OA[c]	Oxford LoE[d]
					T2 median: 44.94<br>T3 median: 34.58<br>T1/T2 p=0.016<br>T2/T3 p=0.044<br>T1/T3 p=0.860		
Solati, 2017 (23)	Intervention: 23<br>Control: 23	P: Attend a mindfulness-based cognitive therapy<br>C: General recommendations about health lifestyle	Other SLE patients	GHQ-28<br>SF-36	Depression<br>MBCT pre: 13.6 (4.1)<br>MBCT post: 8.9 (2.3)<br>CG pre: 12.7 (2.5)<br>CG post: 14.4 (2.8)<br><br>Quality of life<br>MCS=<br>MBCT pre: 43.7 (11.0)<br>MBCT post: 51.6 (10.4)<br>CG pre:44.3 (10.8)<br>CG post: 43.7 (11.5)<br>p<0.050<br>PCS=<br>MBCT pre: 44.0 (11.5)<br>MBCT post: 49.7 (10.6)<br>CG pre: 43.2 (10.4)<br>CG post: 44.3 (11.7)<br>p>0.050<br><br>Anxiety<br>MBCT pre: 13.8 (4.2)<br>MBCT post: 9.2 (3.5)<br>CG pre: 13.4 (3.2)<br>CG post: 14.5 (3.5)<br><br>Social function<br>MBCT pre: 15.6 (3.5)<br>MBCT post: 8.8 (2.7)<br>CG pre: 13.4 (3.7)<br>CG post: 14.7 (4.1)	11<br>I	2
Yelnik, 2017 (24)	Intervention: 121<br>Control: -	P: 3-year CVD prevention counselling program<br>C: N/A: intraindividual assessment	N/A: intraindividual assessment	Blood pressure<br>Blood glucose<br>Cholesterol profile<br>BMI	CVD risk<br>Systolic blood pressure improvement (-6.12 +/- 2.16 mm Hg<br>p<0.05)<br>Prevalence of abnormal cholesterol profile decreased with significant improvements in mean HDL (+5.4 +/- 0.36 mg/dl<br>p<0.0001) and triglyceride levels (-12.6 +/- 5.40 mg/dl<br>p<0.05)	10<br>I	4
Kusnanto, 2018 (25)	Intervention: 36<br>Control: -	P: Session of mentoring<br>C: N/A: intraindividual assessment	N/A: intraindividual assessment	Self-care agency scale<br>Self-rated abilities on the health practices scale<br>Lupus quality inventory	Self-care agency<br>Improved by 19.93%<br><br>Self-care activity<br>Improved by 17.53%<br><br>Quality of life<br>Improved by 12.19%.	10<br>I	4
Scalzi, 2018 (26)	Intervention:13<br>Control: 14	P: Follow a web-based educational program + answer module questions on an online social media forum with other participants<br>C: Usual care	Other SLE patients	MPR (medication possession ratio)<br>MASRI (medication adherence self-report inventory)<br>PSQ<br>CASE (children arthritis self-efficacy scale)	Medication adherence (to HCQ)<br>MPR:<br>SM group: w0: 0.75(0.06)<br>w6: 0.92 (0.03)<br>p<0.001<br>Control group: w0: 0.79 (0.7)<br>w6 =0.81 (0.05)<br>p=0.56<br><br>MASRI:<br>SM group: w0: 85.4 (6.7)<br>w6: 84.2 (7.7)<br>p=0.044	11<br>W	3

Author, year	Participants	Intervention/management strategy[a]	Comparator	Outcome(s)[b]	Results	SD&OA[c]	Oxford LoE[d]
				SMILEY (erythematosus in youngsters) index<br>3 Likert scale questions<br>22-item scale<br>Validated Likert scale	Control group: w0: 87.8 (4.0) w6 = 90.4 (2.4) p=0.76<br><br>Stress<br>PSQ did not improve significantly (p=0.35)<br><br>Self-efficacy<br>CASE<br>SM group: w0: 34.3 (3.4) w6:38.5 (3.4)<br>P value= 0.04<br>Control group: w0: 37.0 (2.9) w6 = 36.6 (2.9) p=0.47<br><br>HRQoL<br>SMILEY did not improve significantly (p=0.06)<br><br>SOA (Self of agency)<br>SM group: w0: 17.0 (1.8) w6: 20.8(1.3)<br>P value = 0.03<br>Control group: w0: 16.2 (1.5) w6 = 17.3 (1.2) P value =0.2<br><br>SOC (Self of community)<br>SM group: w0: 138.3 (13.4) w6: 168.3 (10.2)<br>P value= 0.03<br>Control group: w0: 143.1 (14.0) w6 = 152.2 (11.3) P value =0.4		
Williams, 2018 (27)	Intervention: 23<br>Control: -	P: Receive education and support by a peer-to-peer mentoring<br>C: N/A: intraindividual assessment	N/A: intraindividual assessment	SF-36<br>PHQ-9<br>GAD-7<br>PSS<br>Patient activation measure<br>Systemic Lupus Activity Questionnaire	Quality of life<br>Improved but not statistically significant<br><br>Depression<br>T1:8.28 (0.94)<br>T2: 5.66 (0.96) p=0.057<br><br>Anxiety<br>T1: 7.72 (0.99)<br>T2: 4.2 (1.02) p=0.018<br><br>Stress<br>T1: 8.2 (0.58)<br>T2: 8.28 (0.6) p=0.92<br><br>Disease activity<br>T1:32.36 (5.36) T2:7.66(5.5) p=0.004	10<br>I	4
Kim, 2019 (28)	Intervention: 25<br>Control: -	P: Attend a mindfulness-based cognitive therapy + homework<br>C: N/A: intraindividual assessment	N/A: intraindividual assessment	Korean version of Beck Depression Inventory-II (BDI-II)<br>Beck Anxiety Inventory (BAI)<br>Satisfaction with Life Scale (SWLS)<br>Perceived Stress Scale (PSS)	Depression<br>Pre: 24.6 ± 11.2<br>Post: 17.4 ± 13.0 (p<0.01)<br><br>Anxiety<br>Pre: 18.2 ± 9.5<br>Post: 13.4 ± 7.7 (p=0.04)<br><br>Satisfaction with life<br>Pre: 13.9 ± 6.4<br>Post: 15.4 ± 8.3 (p=0.48)<br><br>Stress<br>Pre: 20.4 ± 3.2<br>Post: 17.9 ± 4.6 (p=0.04)<br><br>Disease activity<br>No improvement	10<br>I	4

Author, year	Participants	Intervention/management strategy[a]	Comparator	Outcome(s)[b]	Results	SD&OA[c]	Oxford LoE[d]
Sahebari, 2019 (29)	Intervention: 12<br>Control: 12	P: Eight Sessions of Acceptance and Commitment Therapy (ACT)<br>C: Usual care	Other SLE patients	Beck's Hopelessness Scale<br>Kessler's Psychological Distress Inventory<br>Krupp's Psychasthenia Inventory	Disappointment<br>Intervention<br>Pre: 7.72 (56.50)<br>Post: 5.28 (36.50)<br>CG<br>Pre: 4.33 (53.75)<br>Post 5.33 (57.83)<br><br>Psychological distress<br>Intervention<br>Pre: 3.27 (26.75)<br>Post: 5.33 (9.42)<br>CG<br>Pre: 2.44 (29.17)<br>Post: 4.33 (29)<br><br>Psychasthenia<br>Intervention<br>Pre: 11.27 (49.17)<br>Post: 8.32 (19.43)<br>CG<br>Pre: 11.14 (45.25)<br>Post: 11.14 (44.25)	10<br>I	3
Williams, 2019 (30)	Intervention: 20<br>Control: -	P: To be enrolled in the Peer Approaches to Lupus Self-management (PALS) program<br>C: N/A: intraindividual assessment	N/A: intraindividual assessment	PHQ-8<br>GAD-8 score<br>SLAQ<br>Th1/Th2 cytokine balance<br>LUP-QOL<br>Patient Activation Measure	Depression<br>changes of 2.62 or 11% change p=0.05<br><br>Anxiety<br>score change of 3.52 or 15% change p=0.018<br><br>Disease activity<br>SLAQ= change score of 24.70 or 25% change p<0.001	10<br>I	4
Kankaya, 2020 (31)	Intervention: 40<br>Control: 40	P: Web-based education programme (3 months) followed by telephone counselling by physicians (3 months)<br>C: Usual care	Other SLE patients	FSS<br>Self-Efficacy for Managing Chronic Disease 6-Item Scale	Fatigue<br>Intervention<br>Pre: 4.5 (1.2)<br>post: 3.9 (1.3)<br>p<0.001<br>Control<br>Pre: 4.7 (1.2)<br>post: 5.0 (1.4)<br>p=0.001<br>P intergroup: 0.001<br><br>Self-efficacy<br>Intervention<br>Pre: 4.6 (2.0)<br>post: 5.2 (1.9)<br>p=0.002<br>Control<br>Pre: 4.5 (2.1)<br>post: 4.3 (2.2)<br>p=0.007<br>P intergroup: 0.04	11<br>W	3
Khan, 2020 (32)	Intervention: 25<br>Control: 22	P: App for self-tracking lifestyle activities and symptoms, and weekly telehealth coaching sessions<br>C: Usual care	Other SLE patients	Number of days with at least 1 login<br>FACIT-Fatigue<br>LupusQoL<br>BPI-SF	Adherence<br>Adherence App: 91.1 (50–9-97.3)<br>Adherence coaching: 81.3 (25.0–81.3)<br><br>Fatigue<br>FACIT-F change from baseline<br>Intervention: 4.0 (-3.5, 21.0)<br>p=0.04<br>Control: -0.5 (-5.0, 7.3)<br>p=0.75<br>P intergroup: 0.17<br><br>HRQoL	11<br>W	3

Author, year	Participants	Intervention/management strategy[a]	Comparator	Outcome(s)[b]	Results	SD&OA[c]	Oxford LoE[d]
					No significant differences between treatment groups in LupusQoL domains in the ITT population.<br><br>Pain<br>BPI Pain severity change from baseline<br>Intervention: 0.0 (-2.8, 2.3) p=0.76<br>Control: 0.6 (-1.3, 1.0) p=0.68<br>P intergroup: 0.73		
Allen, 2021 (33)	Intervention: 30<br>Control: 30	P: PainTRAINER: 8 weeks, automated, internet-based version of pain coping skills training programme<br>C: Usual care	Other SLE patients	Coping strategies questionnaire<br>PROMIS-29<br>LupusPRO<br>PROMIS pain interference instrument	HRQoL<br>Intervention: improvement in sleep disturbance, anxiety/depression, and fatigue, and LupusPRO HRQoL.<br>Control: improvement in fatigue and in LupusPRO HRQoL<br>no changes in other domains.<br><br>Pain<br>Change in pain catastrophizing<br>Intervention: −0.9 (8.9)<br>Control: 2.3 (9.6)	11<br>W	3
White, 2021 (34)	Intervention: 24<br>Control: -	P: Follow the Chronic Disease Self-Management Program (CDSMP)<br>C: Usual care	Other SLE patients	Chew Health Literacy Scale<br>Lupus Self-Efficacy Scale<br>Patient activation measure (PAM)<br>SLAQ	Health literacy<br>No significant differences in pre-post changes between-group comparisons (p=0.82)<br><br>Self-efficacy<br>Self-efficacy: significant increase in mean score for the intervention group p=0.02, but not for the control group p=0.23<br><br>Patient activation<br>Treatment group (p=0.47) and control group (p=0.55).<br><br>Disease activity<br>Not statistically significant change (p=0.37)	11<br>W	3
Xu, 2021 (35)	Intervention: 42<br>Control: 43	P: Receive psychoeducational interventions<br>C: Health education, and nontargeted psychological comfort	Other SLE patients	WHOQOL-BREF<br>BDI<br>STAI	Quality of Life<br>Increase of all four domains of the WHOQOL-BREF at 3 months (p<0:05)<br><br>Depression<br>Reduced, p<0.05<br><br>Anxiety<br>Reduced, p<0.05	11<br>W	3
McCormick, 2022 (36)	Intervention: 83<br>Control: -	P: Acceptance and commitment therapy (ACT)<br>C: N/A	N/A: intraindividual assessment	Anxiety<br>Quality of life	Anxiety<br>Non-significant.<br><br>Quality of life<br>Non-significant.	10<br>W	5
Kang, 2023 (37)	Intervention: 42<br>Control: 42	P: Knowledge, Attitude, and Practice (KAP) model combined with motivational interviewing<br>C: Usual care	Other SLE patients	Health literacy<br>Compliance	Health literacy<br>Improved in intervention group.<br><br>Compliance: Improved in intervention group.	10<br>I	3
Kawka, 2023 (38)	Intervention: 1250<br>Control: -	P: Lupus Expert System for Assessment of Fatigue (LEAF)<br>C: N/A	N/A: intraindividual assessment	Fatigue	Fatigue<br>Not presented.	10<br>W	5

Author, year	Participants	Intervention/management strategy[a]	Comparator	Outcome(s)[b]	Results	SD&OA[c]	Oxford LoE[d]
Pasyar, 2023 (39)	Intervention: 62<br>Control: 62	P: Supportive counseling through smart phone<br>C: Usual care	Other SLE patients	Anxiety	Anxiety<br>Significant improvement in intervention group.	11<br>I	2
Shami, 2023 (40)	Intervention: 46<br>Control: 55	P: Weekly counselling sessions<br>C: Usual care	Other SLE patients	Sexual function	Sexual function<br>Improved in the intervention group.	11<br>I	2
**Physical activity and exercise**							
Ramsey-Goldman, 2000 (41)	Intervention: 5<br>Control: 5	P: Phase I: supervised aerobic exercise at 70-80% of maximum heart rate. Phase II: continue exercise in the supervised setting for 1 month/ unsupervised home exercise programme for 6 months.<br>C: Range of motion/muscle strengthening	Other SLE patients	Exercise treadmill: maximum exercise capacity (METS)<br>Bone mineral density<br>Biomarkers: PTH, osteocalcin<br>SLAM<br>FSS<br>SF-36 PF<br>Maximum muscle strength (isokinetic exercise machine, CYBEX)<br>SF-36	Cardiovascular fitness<br>Exercise (METS)<br>Aerobic: 0.64 (-0.11, 1.39)<br>ROM/MS: 1.25 (0.75, 1.75)<br>p>0.05<br><br>Mineral density<br>BMD %T-score (lumbar) change from baseline<br>Aerobic: -0.20 (-2.26, 1.86)<br>ROM/MS: -5.00 (-15.00, 5.00)<br>p>0.05<br><br>Disease activity<br>SLAM change from baseline<br>Aerobic: 2.80 (0.90, 4.70)<br>ROM/MS: 0.40 (-2.27, 3.07)<br>p>0.05<br><br>Fatigue<br>FSS change from baseline<br>Aerobic: -0.71 (-1.23, -0.18)<br>ROM/MS: -0.68 (-1.22, -0.13)<br>p>0.05<br><br>Isometric strength<br>Both groups showed a significant increase in hamstring but not quadriceps strength. No differences between groups.<br><br>HRQoL<br>SF-36 PCS change from baseline<br>Aerobic: 7.00 (-4.80, 18.80)<br>ROM/MS: 2.5 (-23.11, 28.11)<br>p>0.05	11<br>W	3
Tench, 2003 (42)	Intervention: 62<br>Control: 32	P: Exercise group= exercise (walking, cycling, and swimming)<br>Relaxation group= listen to a relaxation audiotape in a darkened, warm, and quiet room<br>C: Relaxation group: listen to relaxation audiotape	Other SLE patients	HAD<br>SLAM<br>Clinical global impression change score<br>FSS<br>Chalder Fatigue Scale (CFS)<br>VAS fatigue<br>SF-36<br>PSQI<br>Test duration, max O2 uptake, max ventilation, max HR, recovery HR.	Anxiety<br>Exercise: 7.4 (0.8)<br>4.6 (0.7)<br>Control: 8.2 (0.8)<br>5.7 (0.6)<br>p=0.62<br><br>Disease Activity (SLAM)<br>Exercise: 4 (3–8)<br>Control: 6 (4–7). p=0.20<br><br>Fatigue<br>Exercise: 15 (1.5)<br>239 (15)<br>Control: 21 (1.6)<br>283 (14)<br>p=0.05<br><br>HRQoL<br>No significant differences in SF-36 PF, RP, VT.<br><br>Quality of sleep<br>Exercise: 6 (3–9)<br>Control: 8 (5–11). p=0.50	11<br>I	2

Author, year	Participants	Intervention/management strategy[a]	Comparator	Outcome(s)[b]	Results	SD&OA[c]	Oxford LoE[d]
					Aerobic capacity<br>No significant differences in test duration, max O2 uptake, max ventilation, max HR, recovery HR.		
Carvalho, 2005 (43)	Intervention: 41<br>Control: 19	P: Supervised aerobic exercise: incremental load on a treadmill<br>C: Usual care	Other SLE patients	Physiologic variables<br>HAQ<br>SF-36<br>BDI<br>VAS for pain<br>VAS for fatigue	Aerobic capacity<br>Training: Improvement in max exercise tolerance, VO2 max, anaerobic threshold, max ventilation and Borg scale from baseline.<br>Greater improvements than control in anaerobic threshold.<br><br>HRQoL<br>Training: Improvement in all scales but SF-36 BP from baseline.<br>Greater improvements than control in SF-36 PF and VT<br><br>Depression<br>Training: pre: 2.0 (2.7)<br>post: 1.7 (2.7)<br>Control: pre: 2.5 (2.7)<br>post: 3.0 (3.5)<br>P intragroup: 0.47<br>intergroup: 0.10<br><br>Pain<br>Training: pre: 8.4 (12.8)<br>post: 2.9 (3.0)<br>Control: pre: 5.8 (6.4)<br>post: 6.6 (8.5)<br>P intragroup: <0.001<br>intergroup: 0.15<br><br>Fatigue<br>Training: pre: 3.6 (1.5)<br>post: 3.3 (1.3)<br>Control: pre: 3.3 (1.3)<br>post: 3.3 (1.5)<br>P intragroup: <0.001<br>intergroup: 0.10<br><br>HAQ<br>Training: pre: 0.14 (0.21)<br>post: 0.06 (0.19)<br>Control: pre: 0.23 (0.27)<br>post: 0.38 (1.14)<br>P intragroup: 0.01<br>intergroup: 0.03	10<br>I	4
Clarke-Jenssen, 2005 (44)	Intervention: 6<br>Control: -	P: Supervised aerobic exercise: walking on a treadmill for a 3-month programme<br>C: N/A: intraindividual assessment	N/A: intraindividual assessment	SLEDAI<br>Modified HAQ<br>SF-36 VT and BP<br>Aerobic capacity (VO2 max)	Disease activity<br>No significant changes in SLEDAI, CRP and ESR after exercise.<br><br>Aerobic capacity<br>VO2 max increased after exercise compared with baseline (p=0.05)<br><br>HRQoL<br>No significant changes in SF-36 pain score (p=0.1) and MHAQ score (p=0.08).<br><br>Fatigue	10<br>R	4

Author, year	Participants	Intervention/management strategy[a]	Comparator	Outcome(s)[b]	Results	SD&OA[c]	Oxford LoE[d]
					SF-36 VT score improved after exercise compared with baseline (p=0.03)		
do Prado, 2011 (45)	Intervention: 20<br>Control: 20	P: Cardiorespiratory exercise test carried out on a treadmill<br>C: No	Healthy controls	Tidal volume (VT)<br>breathing frequency (BF)<br>total respiratory time (TOT)<br>inspiratory time (TI)<br>expiratory time (TE)<br>inspiratory time to total time (TI/TOT)<br>mean inspiratory flow (VT/TI)<br>ventilatory equivalent for carbon dioxide (VE/VCO2)<br>and end-tidal carbon dioxide pressure (PETCO2)	Ventilatory efficiency<br>BF, BF/VT, VE/VCO2: significantly higher in SLE patients vs controls (p<0.05).<br>VT, TE, TI, TOT, PETCO2: significantly lower in SLE patients vs controls (p<0.05).	1<br>R	3
Otto, 2011 (46)	Intervention: 15<br>Control: -	P: Program of increasing exercise from 100 to 300 min/week (combined with reduced-calorie diet)<br>C: N/A: intraindividual assessment	N/A: intraindividual assessment	BMI<br>Waist circumference<br>Self-reported physical activity	Body composition<br>Weight (kg): - 8.2 (2.0) kg from baseline<br>p<0.05<br>Waist circumference (cm) = -10.8 (4.9) cm from baseline<br>p<0.05<br><br>Physical activity<br>Physical activity (min/session): +25.6 min/session from baseline<br>p<0.05	10<br>W	5
Yuen, 2011 (47)	Intervention: 15<br>Control: -	P: Home exercise program using WII Fit interactive video game for 10 weeks<br>C: N/A: intraindividual assessment	N/A: intraindividual assessment	Body weigh<br>Waist circumference<br>HADS<br>FSS<br>Short-form of the McGill Pain Questionnaire (SF-MPQ) at week 10<br>Pittsburgh Sleep Quality Index (PSQI)	Body composition<br>Weight (kg): baseline: 75.4 (17.4)<br>after Wii: 73.6 (16.9) p=0.01<br>Waist circumference (cm): baseline: 90.8 (16.4)<br>after Wii: 88.0 (15.2) p=0.01<br><br>Anxiety/Depression<br>HADS anxiety: baseline: 8.5 (3.4)<br>after Wii: 7.0 (3.0) p=0.03<br>HADS depression: baseline: 5.9 (3.9)<br>after Wii: 4.6 (2.8) p=0.08<br><br>Fatigue<br>FSS: baseline: 53.9 (7.2)<br>after Wii: 44.0 (11.2) p=0.002<br><br>Pain<br>SF-MPQ total index: baseline: 7.1 (9.5)<br>after Wii: 3.8 (7.7) p=0.06<br>SF-MPQ overall intensity: baseline: 1.0 (1.1)<br>after Wii: 0.4 (0.7) p=0.04<br><br>Sleep	10<br>R	4

Author, year	Participants	Intervention/management strategy[a]	Comparator	Outcome(s)[b]	Results	SD&OA[c]	Oxford LoE[d]
					PSQI: baseline: 9.2 (3.6) after Wii: 4.6 (2.8) p=0.07		
Miossi, 2012 (48)	Intervention: 14 Control: 10	P: Supervised training sessions: 35–40 minutes of resistance, 30 minutes of treadmill aerobic training, and 5 minutes of stretching exercises. C: Usual care	Other SLE patients	Chronotropic reserve (CR); Heart rate recovery (absolute change) at the first minute after exercise (HRR1) at the second minute after exercise (HRR2).	Chronotropic reserve Chronotropic reserve: SLE trained: baseline: 81.3 (15.0) post: 95.4 (9.2). SLE non-trained: baseline: 76.1 (18.1) post: 75.6 (16.6) P intragroup<0.05 P intergroup<0.05.<br><br>Heart rate recovery: SLE trained: baseline: 24.1 (9.8) post: 40.9 (10.3). SLE non-trained: baseline: 25.4 (12.8) post: 26.7 (9.3) P intragroup<0.05 P intergroup<0.05.	11 W	3
da Silva, 2013 (49)	Intervention: 27 Control: 30	P: Ergospirometric test C: No	Healthy controls	Cardiopulmonary exercise test IL-6, IL-10 and TNF-a	Inflammatory markers No differences in IL-6, IL-10 and TNF-a after exercise compared to baseline.	10 I	3
dos Reis-Neto, 2013 (50)	Intervention: 18 Control: 20	P: Supervised walking at a heart rate corresponding to the VT1 threshold. C: Usual care	Other SLE patients	SLEDAI Flow-mediated dilation (FMD) Cardiopulmonary exercise test	Disease activity (SLEDAI) Exercise: baseline: 2.0 (2.1) post: 2.4 (2.3) Control: baseline: 2.4 (2.3) post: 3.1 (5.3) P intragroup=0.196 P intergroup: 0.652<br><br>Vascular function Flow mediated dilation (%) Exercise: baseline: 6.3 (6.7) post: 14.1 (9.1) p=0.006 Control: baseline: 8.4 (8.2) post: 9.4 (5.7) p=0.598<br><br>Nitro-glycerine-mediated dilation Exercise: baseline: 20.9 (6.1) post: 24.3 (7.9) p=0.147 Control: baseline: 26.7 (7.1) post: 26.1 (7.0) p=0.782<br><br>Aerobic capacity Improvement in exercise group, but not in controls, in exercise tolerance (min), maximum speed (km/h) and speed VT1, but not in resting HR, VO2 max, HRmax and VE max.	10 R	3
Barnes, 2014 (51)	Intervention: 14 Control: 12	P: Walking, running, cycling, or use of an elliptical machine. C: No	Other SLE patients, healthy controls	SLAQ CRP, IL-12, ICAM-1 and TNF-α Flow-mediated dilation (FMD).	Disease activity SLAQ global score: Sedentary: 1.5 (0.3) Physically active:0.9 (0.3) p>0.05<br><br>SLAQ numerical rating: Sedentary: 5.6 (0.8) Physically active: 3.2 (0.6) p<0.05	1 I	3

Author, year	Participants	Intervention/management strategy[a]	Comparator	Outcome(s)[b]	Results	SD&OA[c]	Oxford LoE[d]
					SLAQ symptom score:<br>Sedentary: 4.1 (1.3)<br>Physically active: 0.8 (0.4)<br>p<0.05<br>Inflammatory markers<br>CRP:<br>Sedentary: 4.4 (0.9)<br>Physically active: 1.4 (0.4)<br>p<0.05<br><br>sICAM-1:<br>Sedentary: 185.8 (24.9)<br>Physically active: 131.2 (9.9)<br>p<0.05<br><br>No differences between groups in IL-6, IL-10, IL-12 and TNF-a<br>Vascular function<br>FMD:<br>No significant differences between sedentary and physically active SLE patients. However, sedentary SLE patients had lowed FMD than healthy controls [3.6 (1.3) vs. 8.1 (1.2)<br>p<0.05], but not the physically active SLE group (p=0.73).		
Perandini, 2014 (52)	Intervention: 8<br>Control: 10	P: Treadmill walking<br>C: No	Healthy controls	Cardiopulmonary exercise test<br>Weight, BMI<br>SLEDAI<br>FSS<br>IFN-gamma, IL-10, IL-6, TNF-alfa, sTNFR1, and sTNFR2<br>CRP<br>C3 and C4<br>SF-36	Aerobic capacity<br>Improvement in time in ventilatory anaerobic threshold, respiratory compensation point, time to exhaustion and HR peak, but not VO2 peak after exercise programme, compared with baseline.<br><br>Body composition<br>BMI: baseline: 25.2 (2.6)<br>post: 24.8 (2.1). p>0.05<br><br>Disease activity<br>SLEDAI: baseline: 1.3 (1.1)<br>post: 0.9 (1.0). p>0.05<br><br>Fatigue<br>FSS: baseline: 33.4 (14.4)<br>post: 26.4 (10.2). p<0.05<br><br>Inflammatory markers<br>Resting cytokine levels: No differences between exercise and control groups in IFN-gamma, IL-10, IL-6, TNF-a and sTNFR1/2.<br>After a single bout of acute moderate or intense aerobic exercise: lower AUC IL-10 group for exercise vs control group. No differences for other cytokines.<br><br>HRQoL<br>SF-36: No significant differences in any subscale after exercise programme, compared with baseline.	10<br>I	3
Benatti, 2015 (53)	Intervention: 17<br>Control: 16	P: Seven strength exercises for the major muscle groups followed by aerobic exercise on a treadmill.	Other SLE patients, healthy controls	Total cholesterol, HDLc, LDLc, VLD	Lipid profile<br>No significant changes in plasma total/HDL/LDL/VLDL cholesterol,	11<br>W	3

Author, year	Participants	Intervention/management strategy[a]	Comparator	Outcome(s)[b]	Results	SD&OA[c]	Oxford LoE[d]
		C: Usual care		Lc, triglycerides. Composition of the HDL subfractions	insulin or glucose levels after intervention.		
Bogdanovic , 2015 (54)	Intervention: 30<br>Control: 30	P: Aerobic training on a bicycle ergometer, for 6 weeks.<br>C: N/A: intraindividual assessment	N/A: intraindividual assessment	Beck depression inventory (BDI)<br>Fatigue Severity Scale (FSS)<br>SF-36	Depression (BDI)<br>BDI moderate or severe depression<br>Aerobic training: baseline: 70%<br>post: 10%<br>p<0.001<br>Isotonic exercise: baseline: 70%<br>post: 6.6%<br>p<0.001<br><br>Fatigue (FSS)<br>Aerobic training: baseline: 53.6 (6.3)<br>post: 29.2 (7.9)<br>p<0.001<br>Isotonic exercise: baseline: 53.6 (6.3)<br>CG after activity: 29.2 (7.9)<br>p<0.001<br>[the reported values are indeed the same]<br><br>HRQoL (SF-36)<br>BDI moderate or severe depression<br>Aerobic training: baseline: 70%<br>post: 10%<br>p<0.001<br>Isotonic exercise: baseline: 70%<br>post: 6.6%<br>p<0.001	11<br>W	3
Perandini, 2015 (55)	Intervention: 23<br>Control: 10	P: Two single bouts of acute aerobic exercise (moderate and intense) performed in a treadmill<br>C: No	Other SLE patients, healthy controls	INF-γ, IL-10, IL-6, TNF-α and soluble TNF receptors (sTNFR1 and sTNFR2)	Inflammatory markers<br>Higher levels of TNF-a and sTNFR1-2 in patients with inactive vs active disease.<br><br>No significant differences in IFN-gamma, IL-6 or IL-10.<br><br>Changes were transient and reached baseline levels after 24h recovery.	10<br>I	3
Abrahao, 2016 (56)	Intervention: 21<br>Control: 21	P: Walking and bicycle vs free weight and elastic bands exercises 3 times/week for 12 weeks<br>C: Usual care	Other SLE patients	BDI<br>SLEDAI<br>SF-36<br>12-minute walk test	Depression (BDI)<br>Cardiovascular training: baseline: 20.6 (5.3)<br>post: 20.1 (7.1). p>0.05<br>Resistance training: baseline: 19.4 (5.0)<br>post: 17.3 (4.4). p>0.05<br>Control: baseline: 19.1 (5.6)<br>post: 20.1 (5.9). p<0.05<br><br>Disease activity (SLEDAI)<br>Cardiovascular training: baseline: 1.8 (0.6)<br>post: 1.6 (0.9). p>0.05<br>Resistance training: baseline: 1.4 (0.6)<br>post: 1.3 (0.5). p>0.05<br>Control: baseline: 2.3 (1.7)<br>post: 1.2 (0.4). p<0.05<br><br>HRQoL (SF-36)<br>Cardiovascular training: significant improvement in all	11<br>I	2

Author, year	Participants	Intervention/management strategy[a]	Comparator	Outcome(s)[b]	Results	SD&OA[c]	Oxford LoE[d]
					SF-36 subscales. Greater improvements than control in SF-36 RP and VT.<br>Resistance training: significant improvement in all SF-36 subscales but VT.<br><br>Physical function (12-min walk test)<br>Cardiovascular training: baseline: 1020 (225)<br>post: 1406 (257). p<0.05<br>Resistance training: baseline: 911 (172)<br>post: 1140 (173). p<0.05<br>Control: baseline: 936 (169)<br>post: 1068 (187). p<0.05<br>CT vs control: p<.0.001<br>RT vs control: p=0.001		
Avaux, 2016 (57)	Intervention: 15<br>Control: 18	P: Endurance exercises (walking or bicycle) + strengthening exercises (with elastoband or weights for both upper and lower limbs)<br>C: Usual care	Other SLE patients	FSS<br>Physical working capacity (measured at 75% of the predicted maximal heart rate PWC75%/kg)<br>Modified Borg's scale to assess perception of exertion.	Fatigue (FSS)<br>Supervised training: reduction in FSS score at month 3 (p=0.007) and 9 (p=0.003) compared with baseline.<br>Home training reduction in FSS score at month 3 (p=0.003) and 9 (p=0.035) compared with baseline.<br>Control: no significant differences in FSS score at month 3 and 9 compared with baseline.<br>[values not reported only box plots].<br><br>Physical capacity<br>Physical working capacity (PWC75%/kg) and the Borg scale did not improve over time in none of the 3 groups, nor at month 3, neither at month 9.	11<br>W	3
Bostrom, 2016 (58)	Intervention: 18<br>Control: 17	P: 0 to 3 months: high + low-moderate intensity aerobic exercise + education+ individual coaching.<br>4 to 12 months: high + low-moderate intensity aerobic exercise + individual coaching<br>C: Usual care	Other SLE patients	Maximal oxygen uptake (VO2 max)<br>SLEDAI<br>SF-36<br>SDI<br>Self-reported question for physical activity	Aerobic capacity<br>Exercise group: significant improvement in VO2 max, 60% max and 80% max at month 12 compared with baseline.<br>No significant differences between exercise and control groups.<br><br>Disease activity<br>Exercise: baseline 1 (0–8)<br>month 12 4 (2–6)<br>Control: baseline 2 (0–3)<br>month 12 2 (0–5)<br>P: intragroup=0.25<br>intergroup: 0.14.<br><br>HRQoL<br>No significant improvement in SF-36 subscales in the exercise and control groups at month 12 compared with baseline.<br>Exercise group showed a greater improvement in SF-36 MH than control (group x time p value= 0.05)<br><br>Organ Damage	11<br>I	2

Author, year	Participants	Intervention/management strategy[a]	Comparator	Outcome(s)[b]	Results	SD&OA[c]	Oxford LoE[d]
					No significant increase in SDI score in exercise and control groups. No significant differences between groups at month 12.<br><br>Physical activity<br>The frequency of self-reported physical activity at high intensity increased for the exercise and control groups at month 12 compared with baseline.		
Perandini, 2016 (59)	Intervention: 8<br>Control: 4	P: Single bout of acute aerobic exercise performed 72 hours after a cardiopulmonary exercise test to determine VAT and RCP<br>C: No	Other SLE patients, healthy controls	Quantitative PCR array assay of a panel of immune-related genes (altered if fold changes of >2)	Inflammatory markers<br>SLEinactive and HC group=down-regulation of innate immunity genes (IL13-IL2-IL18-CCL5) and TLR-related pathway genes at End-ex (up-regulated at baseline) + up-regulation of JAK/STAT in recovery<br>SLEactive= fewer genes down-regulated related to both innate and adaptive immunity (IL2, IFNG,IL18,IL13,GATA3,CCL4) + fewer genes up-regulated in recovery ,resulting in a less connected network.	10<br>I	3
O'Dwyer, 2017 (60)	6 RCTs and 5 quasi-RCTs	P: Various modalities: aerobic exercise programme, resistance training, multi-component interventions.<br>C: N/A	N/A	Aerobic capacity<br>BDI<br>HADS<br>SLEDAI<br>FSS	Aerobic capacity<br>Exercise groups: significant increase compared with control group (mean difference: = 1.85, 95% CI: (1.12, 2.58), p<0.001)<br><br>Depression<br>Exercise groups: significant decrease compared with control group (mean difference: = –0.40, 95% CI (–0.71, –0.09), p=0.01)<br><br>Disease activity<br>Exercise groups: no significant changes compared with control (mean difference: = 0.01, 95% CI: (–0.54, 0.56), p=0.97)<br><br>Fatigue<br>Exercise groups: significant decrease compared with control group (mean difference: = –0.61, 95% CI: (–1.19, –0.02), p=0.04)	12<br>R	1
Wu, 2017 (61)	3 studies: 2 RCT, 1 quasi-experimental	P: Aerobic exercise (treadmill, walking/cycling/swimming)<br>C: N/A	N/A	FSS<br>SF-36 VT<br>VAS fatigue	Fatigue<br>Exercise groups: significant decrease compared with control group (mean difference: = –0.52, 95% CI: (–0.91, –0.13), p=0.009)	12<br>R	1
Benatti, 2018 (62)	Intervention: 9<br>Control: 10	P: Supervised treadmill aerobic training<br>C: No	Other SLE patients	Cardiopulmonary exercise test<br>Body weight<br>Fat, lean mass<br>24-h dietary recalls<br>Fasting glucose and insulin levels	Aerobic capacity<br>Exercise group: significant increases in VAT, time at RCP, time to exhaustion and HR peak, but not VO2 peak compared with control.<br><br>Body composition<br>Body weight (kg):<br>Exercise: baseline 65.0 (10.5) post: -0.3 (-1.7–1.1)	11<br>W	3

Author, year	Participants	Intervention/management strategy[a]	Comparator	Outcome(s)[b]	Results	SD&OA[c]	Oxford LoE[d]
				Matsuda inde x + insulinogenic index phospho-AMPK Thr 172 assessed through muscle biopsy and western blot	p=0.6<br>Control: baseline 67.6 (8.8)<br>post: 0.2 (-1.2–1.5).<br><br>Fat mass (kg):<br>Exercise: baseline 21.7 (6.5)<br>post: 0.1 (-0.9–1.1)<br>p=0.7<br>Control: baseline 22.8 (4.8)<br>post: -0.2 (-1.3–0.9).<br><br>Food Intake<br>No significant differences in change of caloric or macronutrients intake between exercise and control groups.<br><br>Insulin sensitivity<br>Exercise group: greater decreases in fasting insulin, AUC insulin, HOMA IR and fasting free fatty acids, and greater increases in Matsuda index and fasting glucagon, compared with control.<br>No differences in fasting glucose, AUC glucose, fasting proinsulin and insulinogenic index.		
Middleton, 2018 (63)	Intervention: 7<br>Control: -	P: Hatha yoga classes (deep breathing, relaxation, meditation, poses for strength, flexibility, and balance) + encouragement to home practice for 8 weeks<br>C: N/A: intraindividual assessment	N/A: intraindividual assessment	Personal Journals Interviews	Relaxation<br>Exit interviews (mentions)=<br>benefits: 10<br>feeling of general well-being: 2<br>enjoyment: 3<br>Personal journals (mentions)=<br>benefits:21<br>expectation of future benefits: 7<br>feeling of general well-being: 7	9<br>W	5
Soriano-Maldonado, 2018 (64)	Intervention: 26<br>Control: 32	P: Aerobic exercise on a treadmill<br>C: Guidelines about healthy lifestyle	Other SLE patients	High-sensitivity hsCRP, TFN-alfa, IL-6<br>Oxidative stress markers (MPO)<br>Bruce test<br>Arterial stiffness - pulse wave velocity (PWV)	Inflammatory markers<br>Exercise group: No significant differences in change from baseline in hsCRP, TNF-alfa, IL-6, MPO compared with control.<br><br>Physical fitness<br>Increase in Bruce (min)<br>Exercise: 2.49 (0.44)<br>Control: 0.22 (0.41)<br>p=0.001<br><br>Vascular function<br>Change in Pulse wave velocity (m/s)<br>Exercise: -0.26 (0.14)<br>Control: -0.22 (0.13)<br>p=0.860	10<br>I	3
Timoteo, 2018 (65)	Intervention: 5<br>Control: 9	P: Kinesiotherapy protocol for 4 months<br>C: Usual care	Other SLE patients	BMI and skin folds,<br>Body circumferences<br>Percentage of body fat<br>SF-36<br>Cytokine levels: TNF-a, IL-2, IL-5, IL-6, IL-8, IL-10 (ELISA)	Body composition<br>Kinesiotherapy: no significant changes from baseline in anthropometric measurements.<br>Control: increase in abdominal circumference [baseline: 84.5 (81.5-102.0)<br>post: 85.0 (82.8-102.5)<br>p=0.039], no significant changes from baseline in other anthropometric measurements.<br><br>HRQoL	10<br>R	3

Author, year	Participants	Intervention/management strategy[a]	Comparator	Outcome(s)[b]	Results	SD&OA[c]	Oxford LoE[d]
				CD11b+ and CXCR2+ neutrophils and lymphocytes<br>Flexibility tests<br>10 maximal repetitions test (10 RM)<br>Tinetti gait and balance evaluation test	Kinesiotherapy: Improvement in SF-36 BP<br>no significant changes from baseline in other subscales.<br>Control: no significant changes in any SF-36 subscales.<br><br>Inflammatory markers<br>Kinesiotherapy: no significant changes from baseline in IL-2, IL-5, IL-6, IL-8, IL-10 and TNF-a.<br>Control: significant decrease in IL-5, IL-6 and IL-10.<br><br>Strength and flexibility<br>Kinesiotherapy: Improvement in Wells test (cm)<br>no significant changes from baseline in bench press, leg extension, lying legs curls and stretching test.<br>Control: no significant changes in any strength and flexibility tests.		
da Hora, 2019 (66)	2 RCTs	P: Aerobic exercise<br>C: N/A	N/A	SF-36	HRQoL<br>Exercise groups: significant improvement in SF-36 physical functioning (mean difference: = –9.20, 95% CI (-18.16, -0.23), p=0.04), but not in vitality.	12<br>R	3
Sheikh, 2019 (67)	Intervention: 48<br>Control: 27	P: Walk With Ease (WWE) programme<br>C: Did not complete intervention	Other SLE patients	FACIT-Fatigue<br>VAS fatigue<br>VAS pain<br>Satisfaction survey	Fatigue<br>FACIT-Fatigue: baseline: 27.9 (29.3)<br>post: 32.8 (30.5)<br>ES: 0.16 (-0.24–0.57)<br>VAS Fatigue: baseline: 49.5 (71.0)<br>post: 33.4 (71.0). ES 0.23 (-0.19–0.64)<br><br>Pain<br>VAS Pain: baseline: 41.7 (67.2)<br>post: 33.5 (65.5). ES 0.12 (-0.28–0.53)<br><br>Patient satisfaction<br>97.9% satisfied with programme, 97.9% confidence to continue physical activity, 80.4% increased physical activity.	10<br>I	4
Wu, 2019 (68)	Intervention: 38<br>Control: 38	P: Wearing of pedometer + face-to-face physical activity counselling + follow up phone calls<br>C: Usual care	Other SLE patients	FSS<br>SF-36<br>Pittsburgh Sleep Quality Index (PSQI)<br>Daily steps	Fatigue<br>FSS<br>Counselling: baseline 3.4 (1.5)<br>Control: baseline 3.5 (1.7). T12 vs T10 (group x time): B -0.14 p=0.64<br><br>HRQoL<br>Greater improvements at week 12 in SF-36 VT scores in counselling versus control group<br>no differences for other subscales.<br><br>Sleep<br>PSQI<br>Counselling: baseline 6.6 (3.2)<br>Control: baseline 6.2 (3.3). T12 vs T10 (group x time): B -1.24	11<br>I	2

Author, year	Participants	Intervention/management strategy[a]	Comparator	Outcome(s)[b]	Results	SD&OA[c]	Oxford LoE[d]
					p<0.01<br><br>Physical activity<br>Daily steps<br>Counselling: baseline 5820<br>intervention: 7129<br>Control: baseline 2941<br>intervention: 6227<br>p<0.001		
Gavilan-Carrera, 2020 (69)	Intervention: 26<br>Control: 32	P: Moderate to vigorous intensity aerobic exercise<br>C: Physical activity guidelines and basic nutritional information	Other SLE patients	Bruce submaximal treadmill protocol<br>Beck depression inventory<br>Multidimensional fatigue inventory<br>SF-36<br>Perceived stress scale<br>PSQI	Depressive symptoms<br>BDI change<br>Exercise: -4.03 (1.81)<br>Control: .2.25 (1.59). p=0.475<br><br>Fatigue<br>General fatigue change<br>Exercise: -2.57 (1.07)<br>Control: 0.29 (0.94). p=0.049<br><br>HRQoL<br>PCS change<br>Exercise: 2.83 (2.15)<br>Control: 0.49 (2.06). p=0.448<br>MCS change<br>Exercise: 6.51 (2.63)<br>Control: 2.12 (2.47). p=0.237<br><br>Psychological stress<br>PSS change<br>Exercise: -1.6 (1.2)<br>Control: -1.2 (1.06). p=0.805<br><br>Sleep<br>PSQI change<br>Exercise: -0.63 (0.73)<br>Control: -0.96 (0.66). p=0.744	10<br>I	3
Keramiotou, 2020 (70)	Intervention: 31<br>Control: 27	P: Strengthening and stretching upper limb exercises<br>C: Four sessions of training in alternative methods of performing daily activities, use of aids, joint protection and energy conservation	Other SLE patients	Disability of arm, shoulder and hand (DASH)<br>HAQ<br>LupusQoL<br>Grip and pinch strength<br>Purdue test	DASH<br>Exercise w0: 39.02 (16.10)<br>w12: 21.49 (16.19). p<0.001<br>Control w0: 43.08 (16.39)<br>w12: 38.38 (16.29). p=0.058<br><br>HAQ<br>Exercise w0: 0.81 (0.45)<br>w12: 0.45 (0.45). p<0.001<br>Control w0: 1.10 (0.55)<br>w12: 1.04 (0.49). p=0.420<br><br>HRQoL<br>LupusQoL PH<br>Exercise w0: 56.44 (22.62)<br>w12: 72.95 (21.54). p<0.001<br>Control w0: 51.25 (20.62)<br>w12: 53.33 (22.12). p=0.527<br><br>Physical fitness<br>Exercise group: improvement in grip strength, pinch strength and Purdue test at week 12 compared with baseline (p<0.001 for all).<br>Control group: improvement in Purdue test at week 12 compared with baseline (p=0.001). No differences in other variables.	11<br>W	3
Dionello, 2021 (71)	Intervention: 18<br>Control: 9	P: Whole body vibration exercises (WBVE)<br>C: Whole body vibration exercise	Healthy controls	sEMG of GM (gastrocnemius medialis), VL (Vastus	Muscle activation<br>Increase in 100-200% in muscle activation at 30 Hz compared with 0 Hz	11<br>I	2

Author, year	Participants	Intervention/management strategy[a]	Comparator	Outcome(s)[b]	Results	SD&OA[c]	Oxford LoE[d]
				lateralis), TA(tibialis anterior) muscles	p<0.05 for Vastus lateralis.		
Kao, 2021 (72)	Intervention: 12<br>Control: 11	P: Home-based moderate-intensity aerobic exercise and resistance training.<br>C: Usual care	Other SLE patients	Fitness Index (FI) in 2 km walking test at week 12, Go/no-go test, Stroop task	Physical fitness<br>Fitness index: improvement at 12 weeks in intervention group (p=0.042) but not in the control group (p=1.0)<br>Go/no-go test: improvement in reaction time (p=0.036) and performance index (p=0.024) at 12 weeks in intervention group (p=0.042) but not in the control group.	10<br>I	3
Lopes-Souza, 2021 (73)	Intervention: 11<br>Control: 10	P: Whole body vibration exercises (WBVE)<br>C: Isometry training programme	Other SLE patients	FACIT-F<br>Timed up and go (TUG)<br>HAQ<br>SF-36	Fatigue<br>Mean difference in FACIT-F score: 6.63 (0.64-12.60) p=0.03<br><br>Functional ability<br>No significant changes in TUG scores<br><br>Functional impairment<br>No significant changes in HAQ scores<br><br>HRQoL<br>No significant changes in SF-36 scores	11<br>W	3
Lu, 2021 (74)	5 RCTs	P: Exercise<br>C: N/A	N/A	Quality of Life	Quality of Life<br>Improved physical health and function for participants in exercise groups.	12<br>I	1
Patterson, 2021 (75)	Intervention: 41<br>Control: 184	P: No intervention.<br>Exposure: sedentary behaviour, as per one item from the Rapid Assessment of Physical Activity (RAPA).<br>C: Self-reported physical activity	Other SLE patients	Adjusted Risk of Incident Depression (Incident depr ession= a change in PHQ-8 score from less than 10 at baseline to greater than or equal to 10 during follow-up)<br>SLEDAI<br>SDI<br>Rapid assessment of physical activity (RAPA)	Depression<br>Adjusted Risk of Incident Depression= Intervention group (physically Inactive): 3.88 (95% Confidence interval =1.67, 9.03) Control group (physically Active): 1<br><br>Disease activity<br>SLEDAI= physically Inactive: 2.6(2.7)<br>physically Active: 2.9(3.0)<br><br>Organ damage<br>SDI= physically Inactive: 2.2 (2.1)<br>physically Active: 1.7 (1.9)	5<br>R	3
Hashemi, 2022 (76)	Intervention: 14<br>Control: 10	P: Combined aerobic and anaerobic exercise training<br>C: Usual care	Other SLE patients	Cytokine expression levels	Cytokine expression levels<br>TNF-α, IL2, IL-4, and IL-5 decreased significantly in the intervention group. IL-10, IL-13 and IL-22 significantly increased in the control group. No significant differences were found among the mean serum levels of IFN-γ, IL6, IL-9, IL-17A, IL-17F and IL-21.	11<br>W	3
Frade, 2023 (77)	Intervention: 8<br>Control: 7	P: Telehealth-supervised exercise<br>C: Usual care	Other SLE patients	Fatigue<br>Quality of life	Fatigue<br>Mixed results.<br><br>Quality of life<br>Improved emotional well-being.	10<br>W	4

Author, year	Participants	Intervention/management strategy[a]	Comparator	Outcome(s)[b]	Results	SD&OA[c]	Oxford LoE[d]
Lin, 2023 (78)	Intervention: 40<br>Control: 40	P: Walking exercise<br>C: Routine care	Other SLE patients	Disease activity<br>Quality of life	Disease activity<br>No significant improvements.<br><br>Quality of life<br>Improvement in intervention group.	10<br>I	3
Wohland, 2023 (79)	Intervention: 93<br>Control: -	P: Physical exercise<br>C: N/A	N/A: intraindividual assessment	Fatigue	Fatigue<br>Physical exercise was associated with less fatigue.	1<br>I	3
**Diet and nutrition**							
Shah, 2002 (80)	Intervention: 8<br>Control: 8	P: NCEP Step 2 diet: 30% or less calories from fat (7% from saturated fat, 13% from monounsaturated fat, and 10% from polyunsaturated fat), and < 200 mg of cholesterol per day + maintain their usual level of physical activity.<br>C: Usual care	Other SLE patients	Body weight<br>7-day activity recall QOL (VAS)<br>Blood levels of VLDL, HDL, LDL, TG.<br>3-day food record for food intake	Body composition (body weight)<br>Diet group: w0: 79.4 (8.4)<br>w12: 75.7 (7.4)<br>Control group: 86.2 (24.5(<br>w12: 81.5 (20.3)<br>P intragroup: 0.006<br>P intergroup: 0.50<br><br>HRQoL (VAS)<br>Diet group: w0: 59.4 (7.8)<br>w12: 68.4 (7.8)<br>Control group: 56.3 (15.1)<br>w12: 53.8 (18.2)<br>P intragroup: 0.01<br>P intergroup: 0.05<br><br>Lipid profile<br>Total cholesterol<br>Diet group: w0: 222.4 (24.3)<br>w12: 210.1 (25.4)<br>Control group: 199.3 (49.4)<br>w12: 194.3 (24.1)<br>P intragroup: 0.01<br>P intergroup: 0.40<br><br>LDLc<br>Diet group: w0: 136.4 (23.7)<br>w12: 134 (20.6)<br>Control group: 125.3 (36.9)<br>w12: 119.1 (26.8)<br>P intragroup: 0.80<br>P intergroup: 0.60<br><br>HDLc<br>Diet group: w0: 55.6 (17.4)<br>w12: 53.3 (15.9)<br>Control group: 44.0 (10.8)<br>w12: 49.4 (11.6)<br>P intragroup: 0.09<br>P intergroup: 0.04<br><br>TG<br>Diet group: w0: 151.6 (85.2)<br>w12: 114.6 (30.2)<br>Control group: 150.8 (62.2)<br>w12: 128.0 (43.8)<br>P intragroup: 0.80<br>P intergroup: 0.20<br><br>Nutrient intake<br>Diet group: greater reductions in nutrient intake compared to control group in percentage calories from total fat, SFA, MUFA, and PUFA, and dietary cholesterol	11<br>W	3
Minami, 2003 (81)	Intervention: 7<br>Control: 189	P: No intervention.	Other SLE patients	Lupus Activity Criteria Count<br>SDI	Disease activity	5<br>R	3

Author, year	Participants	Intervention/management strategy[a]	Comparator	Outcome(s)[b]	Results	SD&OA[c]	Oxford LoE[d]
		Dietary nutrients estimated by a semiquantitative food frequency questionnaire<br>C: No			High intake of Vitamin C associated with less active disease (RR: 0.26<br>95% CI: (0.1–0.67), p=0.005).<br><br>No significant associations for other nutrients.<br>Cardiovascular risk<br>Higher intake of vegetable fat was found among patients who developed a vascular event versus those who did not (35.9 versus 30.4 g/day<br>p=0.04).<br><br>No significant differences for other nutrients.		
Duffy, 2004 (82)	Intervention: 40<br>Control: 12	P: 1° group: 3g MaxEPA+ 3mg copper<br>2° group: 3g MaxEPA + placebo copper<br>3° group: 3 mg copper+ placebo oil fish<br>C: Placebo	Other SLE patients	BMI<br>SLAM-R<br>Patient-reported improvement	Body composition<br>No significant changes from baseline in BMI in any of the groups.<br><br>Disease activity<br>SLAM-R<br>Fish oil: w0: 6.12<br>w24: 4.69 p<0.05<br>No fish oil: no significant change from baseline.<br>Copper: no significant change from baseline.<br><br>Patient-reported improvement<br>Fish oil and copper:<br>Improvement: 6/13<br>No changes: 7/13.<br>Placebo: Improvement 1/13<br>No changes: 9/13<br>Worsening: 3/13. p=0.027	11<br>W	3
Shah, 2004 (83)	Intervention: 8<br>Control: 7	P: Counselled to follow the NCEP Step II diet: < 30% of energy as fat and < 7% as saturated fat, and < 200 mg of cholesterol per day 21. Counselled to limit their intake of sodium (< 2400 mg/day) and refined and added sugars and consume 2–3 servings of skim/low fat dairy foods and ≥ 5 servings of fruits and vegetables per day.<br>C: Usual care	Other SLE patients	3-day food record at baseline, 6, 12 weeks<br>Changes in nutrient intakes	Nutrient intake<br>Energy intake<br>Diet group: w0: 1693 (320)<br>w12: 1145 (310)<br>Control group: 1386 (509)<br>w12: 1339 (465)<br>P intragroup: 0.02<br>P intergroup: 0.10<br><br>Vitamin B12<br>Diet group: w0: 2.8 (0.8)<br>w12: 1.6 (1.1)<br>Control group: 2.3 (0.9)<br>w12: 2.6 (0.7)<br>P intragroup: 0.02<br>P intergroup: 0.05<br><br>Sodium<br>Diet group: w0: 2.7 (1.2)<br>w12: 1.7 (0.7)<br>Control group: 1.9 (0.6)<br>w12: 1.9 (0.8)<br>P intragroup: <0.05<br>P intergroup: 0.08<br><br>Haemoglobin levels<br>Diet group: w0: 12.4 (1.1)<br>w12: 12.0 (1.1)<br>Control group: 11.3 (1.8)<br>w12: 10.9 (1.7)<br>P intragroup: >0.05<br>P intergroup: 0.08	11<br>W	3

Author, year	Participants	Intervention/management strategy[a]	Comparator	Outcome(s)[b]	Results	SD&OA[c]	Oxford LoE[d]
Aghdassi, 2010 (84)	Intervention: 137<br>Control: 122	P: Assessing daily use of micronutrient supplements (MS) in SLE patients: Calcium, Vitamin D, Multivitamins (vitamin B6, folic acid, minerals iron, B12, C, E, magnesium, potassium).<br>C: No	Other SLE patients	SLAM- R<br>SLEDAI 2K<br>Use of healthcare resources (visit to healthcare professionals, use of diagnostic tests, hospital emergency visits)<br>SF-36<br>SDI	Healthcare utilisation<br>Compared with non-users, MS users frequently visited health-care professionals and used diagnostics tests.<br><br>Disease activity<br>No difference in SLEDAI-2K or SLAM between users and non-users.<br><br>Lower SLAM in MS users (4.0 (0.4)) versus non-users (5.0 (0.3)) after excluding those taking calcium /vitamin D.<br><br>HRQoL<br>No differences between users and non-users in SF-36 PCS and MCS scores.<br><br>Organ damage<br>Higher SDI in MS users (1.6 (0.2)) versus non-users (1.2 (0.1)).p=0.02	1<br>I	3
Minami, 2011 (85)	Intervention: 216<br>Control: -	P: No intervention. Dietary nutrients estimated by a semiquantitative food frequency questionnaire (Vitamin B6, Vitamin B12, folate, total dietary fibre, soluble dietary fibre, insoluble dietary fibre).<br>C: No	Other SLE patients	Food frequency questionnaire (FFQ)<br>Lupus Activity Criteria Count	Disease activity<br>High intake of Vitamin B6 (HR: 0.41<br>95% CI: 0.18–0.97<br>p=0.04), and total dietary fibre (HR: 0.29, 95% CI: (0.11, 0.78), p=0.01) associated with less active disease<br><br>No significant associations for Vitamin B12 or folate<br>Cardiovascular risk<br>No significant associations between nutrient intake and vascular events.	5<br>R	3
Davies, 2012 (86)	Intervention: 11<br>Control: 12	P: Low GI diet whereby carbohydrate intake was limited to 45 g per day of low GI food, without restricting the consumption of fat and protein<br>C: Low calorie diet	Other SLE patients	Weight, BMI<br>Waist circumference<br>BILAG<br>ECLAM<br>SLEDAI<br>Fatigue Severity Scale<br>PSQI	Body composition<br>Weight loss from baseline (kg)<br>Low GI diet 3.9 (0.9)<br>Low Cal diet 2.4 (2.2) p<0.01<br>P intragroup: <0.001<br>P intergroup: > 0.05<br><br>Disease activity<br>SLEDAI<br>Low GI diet: w0: 3.2 (5.1)<br>w6: 2.8 (6.1)<br>Low Cal diet: w0: 1.0 (1.2)<br>w6: 1.0 (1.2)<br>P intragroup: =0.03<br>P intergroup: >0.05<br>ECLAM<br>Low GI diet: w0: 1.7 (1.2)<br>w6: 1.3 (1.4)<br>Low Cal diet: w0: 1.8 (1.2)<br>w6: 2.2 (1.1)<br>P intragroup: =0.03<br>P intergroup: >0.05<br><br>Fatigue<br>FSS<br>Low GI diet: w0: 4.9 (0.9)<br>w6: 4.4(1.2)<br>Low Cal diet: w0: 4.7(1.5)<br>w6: 4.4(1.7)	11<br>W	3

Author, year	Participants	Intervention/management strategy[a]	Comparator	Outcome(s)[b]	Results	SD&OA[c]	Oxford LoE[d]
					P intragroup: =0.03<br>P intergroup: >0.05<br><br>Seep quality<br>PSQI<br>Low GI diet: w0: 9.3(5.2)<br>w6: 6.7(4.3)<br>Low Cal diet w0: 7.1(4.2)<br>w6: 7.6 (4.7)<br>P intragroup: >0.05<br>P intergroup: >0.05		
Elkan, 2012 (87)	Intervention: 114<br>Control: 122	P: No intervention. Administration of food frequency questionnaire (FFQ) + study of fatty acid content and plaque occurrence<br>C: No	Other SLE patients	Food frequency questionnaire (FFQ)<br>SLAM<br>SLEDAI<br>Intima-media thickness (IMT)<br>Occurrence of plaque<br>SDI	Cardiovascular risk<br>Omega-3 (r = -0.20, p=0.049), EPA (r = -0.32, p=0.002) and DHA (r = -0.33, p=0.001) in adipose tissue correlated negatively with plaque presence, whereas Omega-6 (r = 0.22, p=0.027) and linoleic acid (r = 0.24, p=0.019) correlated positively.<br>Disease activity<br>EPA (r= -0.36, p<0.001) and DHA (r= -0.33, p<0.001) in adipose tissue correlated negatively with SLEDAI<br>Organ damage<br>Arachidonic acid in adipose tissue correlated positively with SDI (r= -0.20, p<0.005).	1<br>I	3
Khajehdehi, 2012 (88)	Intervention: 12<br>Control:12	P: With each meal, each patient received 1 capsule for 3 months, containing 500 mg turmeric (22.1 mg was the active ingredient curcumin)<br>C: Placebo	Other SLE patients	Haematuria<br>Proteinuria<br>Systolic blood pressure<br>C3, C4, anti-dsDNA	Renal function<br>Proteinuria (mg/day)<br>Turmeric: m0: 954.2 (836.6)<br>m3: 260.9 (106.2).<br>Placebo: m0: 527.7 (388.3)<br>m3: 471.4 (292.3).<br>P intragroup: 0.009<br>P intergroup>0.05<br><br>Systolic blood pressure (mmHg)<br>Turmeric: m0: 13.3 (2.3)<br>m3: 12.4 (1.8).<br>Placebo: m0: 12.5 (2.4)<br>m3: 12.3 (1.5).<br>P intragroup: 0.02<br>P intergroup>0.05<br><br>Haematuria<br>Significant decrease from baseline in RBC in the turmeric (p=0.02) but not in the placebo group.<br><br>No significant differences in diastolic blood pressure, GFR, serum albumin.<br><br>Inflammatory markers<br>Turmeric:<br>C4: m0: 17.0 (8.5)<br>m3: 22.9 (9.0)<br>p=0.02.<br><br>No significant changes from baseline in C3 and anti-dsDNA levels.<br>No significant differences between groups changes from baseline in C4, C4 and anti-	11<br>I	2

Author, year	Participants	Intervention/management strategy[a]	Comparator	Outcome(s)[b]	Results	SD&OA[c]	Oxford LoE[d]
					dsDNA levels in the turmeric group.		
Everett, 2015 (89)	Intervention: 41<br>Control: 30	P: CVD-PCP counselling program= Phase 1: assessment of CVD risk factor on patients<br>Phase 2: education on cardiovascular diseases and discussion on prevention strategies. Followed by a patient-centred nutrition counselling to attend at least once a month<br>C: Usual care	Other SLE patients	Weight, BMI<br>Waist circumference<br>Changes in nutrient intake	Body composition<br>Weight (kg): m0: 86.0 (20.2)<br>m6: 84.3 (19.0)<br>p=0.025.<br>BMI: m0: 31.3 (7.4)<br>m6: 30.9 (7.2)<br>p=0.07.<br>Waist circumference (cm): m0: 101.3 (15.1)<br>m6: 102.3 (14.2)<br>p=0.37.<br><br>Nutrient intake<br>Total calories: -164.7 kcal at month 6 (p=0.071)<br>%. Calories from fat: -4.13% at month 6 (p=0.011)<br>Sodium: -508.3 mg at month 6 (p=0.006)<br><br>No differences in cholesterol, omega-3/6, fibre, sugar and folate levels.<br>Changes in diet habits: richer in fruits and vegetables (O<0.001), richer in fibre (O=0.011), low-cholesterol diet (p=0.034).	10<br>I	4
Shamekhi, 2017 (90)	Intervention: 32<br>Control: 36	P: 1000 mg of green tea extract<br>C: Placebo	Other SLE patients	SLEDAI<br>SF-36	Disease activity (SLEDAI)<br>Green tea extract: m0: 4.7 (3.3)<br>m3: 2.8 (3.2)<br>Placebo: m0: 3.2 (3.2)<br>m3: 2.9 (3.2)<br>P intragroup: 0.001<br>P intergroup: 0.004.<br><br>HRQoL (SF-36)<br>Green tea extract: significant improvement from baseline in PF, RP, GH and VT.<br>Higher improvements than placebo in PF, GH and VT.	11<br>I	2
Rothman, 2018 (91)	Intervention: 20<br>Control: 20	P: Health coaching (weekly calls to educate and implement changes based on data analysis)<br>C: Usual care	Other SLE patients	FACIT-Fatigue<br>Brief Pain Inventory-Short form<br>LupusQoL	Pain, Fatigue, HRQoL<br>78% improved in the experimental group and 36% in the control group (p<0.01)	11<br>W	3
Pocovi-Gerardino, 2021 (92)	Intervention: 143<br>Control: 16	P: No intervention.<br>Good adherence (>10 points) to Med Diet (14-item questionnaire on food consumption frequency and habits)<br>C: No	Other SLE patients	BMI, fat percentage<br>Lipid profile<br>Ankle-brachial index, BP<br>Comorbidities: T2DM, AHT, dyslipidaemia.<br>hsCRP<br>Homocysteine<br>SLEDAI<br>Anti-dsDNA, C3, C4<br>SDI	Body composition<br>Patients with high adherence had lower frequency of obesity than patients with low adherence (20.7% versus 37.5% p=0.026), a lower mean BMI (25.9 [5.4] versus 31.5 [7.9], p=0.001) and lower fat mass BMI (32.8 [9.0] versus 38.5 [7.9], p=0.002)<br><br>Cardiovascular risk<br>Inverse relationship between Med Diet score and hsCRP: delta= -0.055, 95% CI: (-0.108, -0.003), p=0.039<br>No significant relationship with homocysteine levels.<br>Patients with high adherence had lower concentrations of TG than patients with low adherence, but there were no	1<br>R	3

Author, year	Participants	Intervention/management strategy[a]	Comparator	Outcome(s)[b]	Results	SD&OA[c]	Oxford LoE[d]
					differences in total/HDL/LDL cholesterol.<br><br>Disease activity<br>Inverse relationship between Med Diet score and SLEDAI: beta= -0.380, 95% CI: (-0.464, -0.296), p<0.001<br><br>Organ damage<br>Inverse relationship between Med Diet score and SDI: beta= -0.740, 95% CI: (-0.938, -0.542), p<0.001		
Gwinnutt, 2022 (93)	11 studies	P: Dietary adjustments<br>C: N/A	N/A	Disease activity	Disease activity<br>Evidence for fish oil/omega-3 showed no effect on outcomes.	12<br>I	1
Knippenberg, 2022 (94)	Intervention: 376<br>Control: -	P: Dietary adjustments<br>C: N/A	N/A: intraindividual assessment	Symptom severity	Symptom severity<br>Increased vegetable intake and/or decreased intake of processed food, sugar, gluten, dairy and carbohydrates was associated with lower symptom severity ratings.	1<br>W	4
**Reduction of harmful exposures**							
Stege, 2000 (95)	Intervention: 11<br>Control: -	P: 3 different sunscreens: Sunscreen A: UVB: Octocrylene. UVA: Mexoryl SX, Mexoryl XL, Parsol 1789. TiO2), SPF >60<br>Sunscreen B: (UVB: Eusolex 6300, Parsol MCX, Uvinul T150, Neohelipan. UVA: Parsol 1789. TiO2), SPF >75<br>Sunscreen C: (Eusolex 6300, Parsol MCX, Uvinul T150 UVA: Parsol 1789. TiO2) SPF= 35]<br>C: N/A: intraindividual assessment	N/A: intraindividual assessment	Photoprovocation test<br>Skin biopsies after treatment<br>Semiquantitative RT-PCR for ICAM-1 mRNA expression	Photoprotection<br><br>Complete Photoprotection<br>Sunscreen A in 11 out of 11 patients<br>Sunscreen B in 5 out of 11 patients<br>Sunscreen C in 3 out of 11 patients<br><br>Expression of keratinocyte ICAM-1 mRNA<br><br>Increased in unprotected irradiated skin area<br>not increased in area treated by sunscreen A	10<br>R	4
Herzinger, 2004 (96)	Intervention: 66<br>Control: -	P: 2 mg/cm2 sunscreen Anthelios W30 La Roche-Posay (parsol 1789, uvinul N539, uvinul T150, mexoryl XL, titanium dioxide)<br>C: N/A: intraindividual assessment	N/A: intraindividual assessment	Photoprovocation test<br>Photosensitivity, skin lesions	Photoprotection<br><br>Patients with photosensitivity who were treated with sunscreen developed:<br>LE lesion: 2 (4)<br>No reaction: 26 (47)<br>Pigmentation only: 25 (49)	5<br>W	4
Zahn, 2014 (97)	Intervention: 20<br>Control: 10	P: Broad-spectrum liposomal sunscreen 20 min prior to a combined standardized UVA/UVB irradiation<br>C: Unprotected skin, sunscreen use	Healthy controls, intraindividual assessment	Immunohistological analysis for immune cell subpopulations.	Inflammatory markers<br>MxA: sunscreen reduced MxA positive cells compared with unprotected skin.<br><br>Immune cells: sunscreen reduced the number of CD11c (DC), CD23 (pDC) and CD68 (macrophages) cells compared with unprotected skin.	10<br>I	3
Squance, 2015 (98)	Intervention: 80<br>Control: 41	P: Completing a Home Cleaning and Maintenance Product list (HCMPL) questionnaire<br>C: No	Other SLE patients	SRF (Self-reported flare) risk	Disease flares<br>Bath oil use (IRR 1.008, 95% CI: (1.00, 1.02)) = significant association with increased SRF day relative risk (IRR).<br>Cleansing beauty (IRR 0.999, 95% CI: (0.998, 0.999))	2<br>I	4

Author, year	Participants	Intervention/management strategy[a]	Comparator	Outcome(s)[b]	Results	SD&OA[c]	Oxford LoE[d]
					make-up (IRR 0.998, 95% : (0.997, 0.999)) adhesives (IRR 0.994, 95% CI: (0.991, 0.997)) paint (IRR 0.99, 95% CI: (0.986, 0.995)) = paradoxical "protective" effects (reduced SRF days)		
Xu, 2015 (99)	Intervention: 65 Control: 665	P: No intervention. Self-reported smoking status (smoker: one cigarette per day for three consecutive months) C: No	Other SLE patients	SLEDAI (score and domains) Autoantibodies (dsDNA, anti-Smith, anti-SSA/Ro, anti-SSB/La, anti-ribonucleoprotein (RNP) and anti-ribosomal RNP, APL	Disease activity/manifestations SLEDAI Smokers:12.5(8.9) Non-smokers:10. (7.1) p=0.028 <br>Manifestations Smokers had higher frequency of Microscopic haematuria (30.8% versus19.1%, p=0.025), photosensitivity (35.9% versus 18%, p=0.006), nephropathy (59.4% versus 39.8%, p=0.011) and proteinuria (54.7% versus NS: 35.2%, p=0.010), but not in other SLEDAI descriptors. <br>Inflammatory markers There were no differences between smokers and non-smokers in ANA, anti-dsDNA, anti-Sm, anti-RNP, SSA/SSB and aPL antibody positivity.	1 R	3
Abdul Kadir, 2018 (100)	Intervention: 205 Control: 17	P: Photoprotection awareness C: No	Other SLE patients	SLEDAI-2K SDI Anti-dsDNA, ANA, Ro, La, ENA C3, C4 ESR	Disease activity SLEDAI Aware: 2.0 (4) unaware: 2.0 (3) p=0.41 <br>Organ damage <br>SDI Aware: 1.0 (2) unaware: 1.0 (2) p=0.81 <br>Inflammatory markers No differences in ANA, Ro/La and anti-DSDNA positivity, and in C3/C4 levels.	1 I	3
Raymond, 2021 (101)	Intervention: 99 Control: -	P: Smoking C: N/A	N/A: intraindividual assessment	Serum cytokine levels	Serum cytokine levels Smoking was associated with increased levels of B-cell activating factor and decreased levels of interferon-γ.	1 I	3
**Social relations**							
Li, 2019 (102)	Intervention: 200 Control: -	P: No intervention (exposure to illness uncertainty, social support, coping modes through questionnaires)- being hospitalized for over a week C: N/A: intraindividual assessment	N/A: intraindividual assessment	Mishel Uncertainty in Illness Scale (MUIS) Social Support Rating Scale (SSRS) Medical Coping Modes Questionnaires (MCMQ)	Illness uncertainty Mean: 97.60 ± 11.24 <br>Social support Illness uncertainty negatively correlated with support availability (r = -0.161) <br>Coping modes Illness uncertainty positively correlated with the yielding coping mode (r = 0.249)	1 R	3

[a]P: Intervention/management strategy applied to population under investigation; C: Intervention/management strategy applied to participants in the comparator group. [b]Outcomes from the studies cited in the systematic review: AIMS2: Arthritis Impact Measurement Scales 2; BDI: Beck Depression Index; BMI: Body Mass Index; CES-D: Centre for Epidemiologic Studies Depression Scale; CFS: Chalder Fatigue Scale; CRP: C-Reactive Protein; FACIT-F: Functional Assessment of Chronic Illness Therapy – Fatigue Scale; FSS: Fatigue Severity Scale; GAD-7: Generalized Anxiety Disorder 7-item scale; GHQ-28: General Health Questionnaire-28; HADS: Hospital Anxiety and Depression Scale; HAQ: Health Assessment Questionnaire; PHQ-9: Patient Health Questionnaire 9; PSQ: Pain and Sleep Questionnaire; PSQI: Pittsburgh Sleep Quality Index; SDI: Systemic Lupus International Collaborating Clinics/American College of Rheumatology Damage Index; SF-36: Short-Form Health Survey-36; SLAM: Systemic Lupus Activity Measure; SLAQ: Systemic Lupus Activity Questionnaire; SLEDAI: Systemic Lupus erythematosus Disease Activity Index; STAI: State-Trait Anxiety Inventory. [c]Study design and overall appraisal (adapted from the Joanna Briggs Institute Manual for Evidence Synthesis (103)) [d]LoE: Levels of evidence according to the Oxford Centre for Evidence-Based Medicine (104)

**Notation**	**Study design**
**1**	Analytical cross-sectional study
**2**	Case-control study
**3**	Case report
**4**	Case series
**5**	Cohort study
**6**	Diagnostic test accuracy study
**7**	Economic evaluation
**8**	Prevalence study
**9**	Qualitative research
**10**	Quasi-experimental study
**11**	Randomised controlled trial
**12**	Meta-analysis, with or without systematic review

Notation	Overall appraisal
R	Robust
I	Intermediate
W	Weak

References

1. Sohng KY. Effects of a self-management course for patients with systemic lupus erythematosus. Journal of Advanced Nursing. 2003;42(5):479-86.
2. Brown SJ, Somerset ME, McCabe CS, McHugh NJ. The impact of group education on participants' management of their disease in lupus and scleroderma. Musculoskeletal Care. 2004;2(4):207-17.
3. Dorsey RR, Andresen EM, Moore TL. Health-related quality of life and support group attendance for patients with systemic lupus erythematosus. JCR: Journal of Clinical Rheumatology. 2004;10(1):6-9.
4. Greco CM, Rudy TE, Manzi S. Effects of a stress-reduction program on psychological function, pain, and physical function of systemic lupus erythematosus patients: a randomized controlled trial. Arthritis & Rheumatism. 2004;51(4):625-34.
5. Karlson EW, Liang MH, Eaton H, Huang J, Fitzgerald L, Rogers MP, et al. A randomized clinical trial of a psychoeducational intervention to improve outcomes in systemic lupus erythematosus. Arthritis & Rheumatism. 2004;50(6):1832-41.
6. Goodman D, Morrissey S, Graham D, Bossingham D. The application of cognitive-behaviour therapy in altering illness representations of systemic lupus erythematosus. Behaviour Change. 2005;22(3):156-71.
7. Harrison MJ, Morris KA, Horton R, Toglia J, Barsky J, Chait S, et al. Results of intervention for lupus patients with self-perceived cognitive difficulties. Neurology. 2005;65(8):1325-7.
8. Haupt M, Millen S, Janner M, Falagan D, Fischer-Betz R, Schneider M. Improvement of coping abilities in patients with systemic lupus erythematosus: a prospective study. Annals of the Rheumatic Diseases. 2005;64(11):1618-23.
9. Miljeteig K, Graue M. Evaluation of a multidisciplinary patient education program for people with systemic lupus erythematosus. Journal of Nursing & Healthcare of Chronic Illnesses. 2009;1(1):87-95.
10. Navarrete-Navarrete N, Peralta-Ramirez MI, Sabio-Sanchez JM, Coin MA, Robles-Ortega H, Hidalgo-Tenorio C, et al. Efficacy of cognitive behavioural therapy for the treatment of chronic stress in patients with lupus erythematosus: a randomized controlled trial. Psychotherapy & Psychosomatics. 2010;79(2):107-15.
11. Navarrete-Navarrete N, Peralta-Ramirez MI, Sabio JM, Martinez-Egea I, Santos-Ruiz A, Jimenez-Alonso J. Quality-of-life predictor factors in patients with SLE and their modification after cognitive behavioural therapy. Lupus. 2010;19(14):1632-9.
12. Brown RT, Shaftman SR, Tilley BC, Anthony KK, Kral MC, Maxson B, et al. The health education for lupus study: a randomized controlled cognitive-behavioral intervention targeting psychosocial adjustment and quality of life in adolescent females with systemic lupus erythematosus. American Journal of the Medical Sciences. 2012;344(4):274-82.
13. Drenkard C, Dunlop-Thomas C, Easley K, Bao G, Brady T, Lim SS. Benefits of a self-management program in low-income African-American women with systemic lupus erythematosus: results of a pilot test. Lupus. 2012;21(14):1586-93.
14. Ganachari MS, Almas SA. Evaluation of clinical pharmacist mediated education and counselling of systemic lupus erythematosus patients in tertiary care hospital. Indian Journal of Rheumatology. 2012;7(1):7-12.

15. Zhang J, Wei W, Wang CM. Effects of psychological interventions for patients with systemic lupus erythematosus: a systematic review and meta-analysis. Lupus. 2012;21(10):1077-87.
16. Bantornwan S, Watanapa WB, Hussarin P, Chatsiricharoenkul S, Larpparisuth N, Teerapornlertratt T, et al. Role of meditation in reducing sympathetic hyperactivity and improving quality of life in lupus nephritis patients with chronic kidney disease. Journal of the Medical Association of Thailand. 2014;97:S101-7.
17. Jolly M, Peters KF, Mikolaitis R, Evans-Raoul K, Block JA. Body image intervention to improve health outcomes in lupus: a pilot study. JCR: Journal of Clinical Rheumatology. 2014;20(8):403-10.
18. Liang H, Tian X, Cao LY, Chen YY, Wang CM. Effect of psychological intervention on healthrelated quality of life in people with systemic lupus erythematosus: A systematic review. International Journal of Nursing Sciences. 2014;1(3):298-305.
19. Williams EM, Penfield M, Kamen D, Oates JC. An Intervention to Reduce Psychosocial and Biological Indicators of Stress in African American Lupus Patients: The Balancing Lupus Experiences with Stress Strategies Study. Open J Prev Med. 2014;4(1):22-31.
20. Williams EM, Bruner L, Penfield M, Kamen D, Oates JC. Stress and Depression in Relation to Functional Health Behaviors in African American Patients with Systemic Lupus Erythematosus. Rheumatology (Sunnyvale). 2014;2014(Suppl 4).
21. Horesh D, Glick I, Taub R, Agmon-Levin N, Shoenfeld Y. Mindfulness-based group therapy for systemic lupus erythematosus: A first exploration of a promising mind-body intervention. Complementary Therapies in Clinical Practice. 2017;26:73-5.
22. O'Riordan R, Doran M, Connolly D. Fatigue and Activity Management Education for Individuals with Systemic Lupus Erythematosus. Occupational Therapy International. 2017;2017:4530104.
23. Solati K, Mousavi M, Kheiri S, Hasanpour-Dehkordi A. The Effectiveness of Mindfulness-based Cognitive Therapy on Psychological Symptoms and Quality of Life in Systemic Lupus Erythematosus Patients: A Randomized Controlled Trial. Oman Medical Journal. 2017;32(5):378-85.
24. Yelnik CM, Richey M, Haiduc V, Everett S, Zhang M, Erkan D. Cardiovascular Disease Prevention Counseling Program for Systemic Lupus Erythematosus Patients. Arthritis care & research. 2017;69(8):1209-16.
25. Kusnanto K, Sari N, Harmayetty H, Efendi F, Gunawan J. Self-care model application to improve self-care agency, self-care activities, and quality of life in patients with systemic lupus erythematosus. Journal of Taibah University Medical Sciences. 2018;13(5):472-8.
26. Scalzi LV, Hollenbeak CS, Mascuilli E, Olsen N. Improvement of medication adherence in adolescents and young adults with SLE using web-based education with and without a social media intervention, a pilot study. Pediatric Rheumatology Online Journal. 2018;16(1):18.
27. Williams EM, Hyer JM, Viswanathan R, Faith TD, Voronca D, Gebregziabher M, et al. Peer-to-Peer Mentoring for African American Women With Lupus: A Feasibility Pilot. Arthritis care & research. 2018;70(6):908-17.
28. Kim HA, Seo L, Jung JY, Kim YW, Lee E, Cho SM, et al. Mindfulness-based cognitive therapy in Korean patients with systemic lupus erythematosus: A pilot study. Complementary Therapies in Clinical Practice. 2019;35:18-21.
29. Sahebari M, Asghari Ebrahimabad MJ, Ahmadi Shoraketokanlo A, Aghamohammadian Sharbaf H, Khodashahi M. Efficacy of Acceptance and Commitment Therapy in Reducing Disappointment, Psychological Distress, and Psychasthenia among Systemic Lupus Erythematosus (SLE) Patients. Iranian Journal of Psychiatry. 2019;14(2):130-6.
30. Williams EM, Dismuke CL, Faith TD, Smalls BL, Brown E, Oates JC, et al. Cost-effectiveness of a peer mentoring intervention to improve disease self-management practices and self-efficacy among African American women with systemic lupus erythematosus: analysis of the Peer Approaches to Lupus Self-management (PALS) pilot study. Lupus. 2019;28(8):937-44.
31. Kankaya H, Karadakovan A. Effects of web-based education and counselling for patients with systemic lupus erythematosus: self-efficacy, fatigue and assessment of care. Lupus. 2020;29(8):884-91.
32. Khan F, Granville N, Malkani R, Chathampally Y. Health-Related Quality of Life Improvements in Systemic Lupus Erythematosus Derived from a Digital Therapeutic Plus Tele-Health Coaching Intervention: Randomized Controlled Pilot Trial. J Med Internet Res. 2020;22(10):e23868.
33. Allen KD, Beauchamp T, Rini C, Keefe FJ, Bennell KL, Cleveland RJ, et al. Pilot study of an internet-based pain coping skills training program for patients with systemic Lupus Erythematosus. BMC Rheumatol. 2021;5(1):20.
34. White AA, Ba A, Faith TD, Ramakrishnan V, Dismuke-Greer CL, Oates JC, et al. The Care-coordination Approach to Learning Lupus Self-Management: a patient navigator intervention for systemic lupus inpatients. Lupus Science & Medicine. 2021;8(1).
35. Xu HY, Teng Q, Zeng Y, Tian CP, Yang BW, Yao XL. Psychoeducational Intervention Benefits the Quality of Life of Patients with Active Systemic Lupus Erythematosus. Journal of Nanomaterials. 2021;2021.
36. McCormick EM, Englund TR, Cleveland RJ, Dickson TA, Schiller CE, Sheikh SZ. ACT for Lupus: Pilot Feasibility and Acceptability Study of a Novel Web-Based Acceptance and Commitment Therapy Program for Patients With Lupus. ACR Open Rheumatol. 2022;4(7):574-80.
37. Kang J, Zhu X, Kan Y, Zhuang S. Application of the Knowledge, Attitude, and Practice model combined with motivational interviewing for health education in female patients with systemic lupus erythematosus. Medicine (Baltimore). 2023;102(12):e33338.
38. Kawka L, Sarmiento-Monroy JC, Mertz P, Pijnenburg L, Rinagel M, Ugarte-Gil MF, et al. Assessment and personalised advice for fatigue in systemic lupus erythematosus using an innovative digital tool: the Lupus Expert system for the Assessment of Fatigue (LEAF) study. RMD Open. 2023;9(4).
39. Pasyar N, Sam A, Rivaz M, Nazarinia M. A smartphone-based supportive counseling on health anxiety and acceptance of disability in Systemic Lupus Erythematosus patients: A randomized clinical trial. Patient Educ Couns. 2023;110:107676.
40. Shami M, Montazeri A, Faezi ST, Behboodi Moghadam Z. The Effect of Sexual Counseling Based on EX-PLISSIT Model on Improving the Sexual Function of Married Women with Systemic Lupus Erythematosus: A Randomized Controlled Trial. Sex Disabil. 2023;41(2):451-66.
41. Ramsey-Goldman R, Schilling EM, Dunlop D, Langman C, Greenland P, Thomas RJ, et al. A pilot study on the effects of exercise in patients with systemic lupus erythematosus. Arthritis Care & Research. 2000;13(5):262-9.
42. Tench CM, McCarthy J, McCurdie I, White PD, D'Cruz DP. Fatigue in systemic lupus erythematosus: a randomized controlled trial of exercise. Rheumatology. 2003;42(9):1050-4.
43. Carvalho MR, Sato EI, Tebexreni AS, Heidecher RT, Schenkman S, Neto TL. Effects of supervised cardiovascular training program on exercise tolerance, aerobic capacity, and quality of life in patients with systemic lupus erythematosus. Arthritis & Rheumatism. 2005;53(6):838-44.
44. Clarke-Jenssen AC, Fredriksen PM, Lilleby V, Mengshoel AM. Effects of supervised aerobic exercise in patients with systemic lupus erythematosus: a pilot study. Arthritis & Rheumatism. 2005;53(2):308-12.
45. do Prado DM, Gualano B, Miossi R, Lima FR, Roschel H, Borba E, et al. Erratic control of breathing during exercise in patients with systemic lupus erythematosus: a pilot-study. Lupus. 2011;20(14):1535-40.
46. Otto AD, Mishler AE, Shah N, Krug MM, Phillips A, Wilson N, et al. Feasibility of Implementing a Lifestyle Intervention in Overweight and Obese Patients with Systemic Lupus Erythematosus. Medicine & Science in Sports & Exercise. 2011;43:123-.
47. Yuen HK, Holthaus K, Kamen DL, Sword DO, Breland HL. Using Wii Fit to reduce fatigue among African American women with systemic lupus erythematosus: a pilot study. Lupus. 2011;20(12):1293-9.
48. Miossi R, Benatti FB, Luciade de Sa Pinto A, Lima FR, Borba EF, Prado DM, et al. Using exercise training to counterbalance chronotropic incompetence and delayed heart rate recovery in systemic lupus erythematosus: a randomized trial. Arthritis care & research. 2012;64(8):1159-66.
49. da Silva AE, dos Reis-Neto ET, da Silva NP, Sato EI. The effect of acute physical exercise on cytokine levels in patients with systemic lupus erythematosus. Lupus. 2013;22(14):1479-83.

50. dos Reis-Neto ET, da Silva AE, Monteiro CM, de Camargo LM, Sato EI. Supervised physical exercise improves endothelial function in patients with systemic lupus erythematosus. Rheumatology. 2013;52(12):2187-95.
51. Barnes JN, Nualnim N, Dhindsa M, Renzi CP, Tanaka H. Macro- and microvascular function in habitually exercising systemic lupus erythematosus patients. Scandinavian Journal of Rheumatology. 2014;43(3):209-16.
52. Perandini LA, Sales-de-Oliveira D, Mello SB, Camara NO, Benatti FB, Lima FR, et al. Exercise training can attenuate the inflammatory milieu in women with systemic lupus erythematosus. Journal of Applied Physiology. 2014;117(6):639-47.
53. Benatti FB, Miossi R, Passareli M, Nakandakare ER, Perandini L, Lima FR, et al. The effects of exercise on lipid profile in systemic lupus erythematosus and healthy individuals: a randomized trial. Rheumatology International. 2015;35(1):61-9.
54. Bogdanovic G, Stojanovich L, Djokovic A, Stanisavljevic N. Physical Activity Program Is Helpful for Improving Quality of Life in Patients with Systemic Lupus Erythematosus. Tohoku Journal of Experimental Medicine. 2015;237(3):193-9.
55. Perandini LA, Sales-de-Oliveira D, Mello S, Camara NO, Benatti FB, Lima FR, et al. Inflammatory cytokine kinetics to single bouts of acute moderate and intense aerobic exercise in women with active and inactive systemic lupus erythematosus. Exercise Immunology Review. 2015;21:174-85.
56. Abrahao MI, Gomiero AB, Peccin MS, Grande AJ, Trevisani VF. Cardiovascular training vs. resistance training for improving quality of life and physical function in patients with systemic lupus erythematosus: a randomized controlled trial. Scandinavian Journal of Rheumatology. 2016;45(3):197-201.
57. Avaux M, Hoellinger P, Nieuwland-Husson S, Fraselle V, Depresseux G, Houssiau FA. Effects of two different exercise programs on chronic fatigue in lupus patients. Acta Clinica Belgica. 2016;71(6):403-6.
58. Bostrom C, Elfving B, Dupre B, Opava CH, Lundberg IE, Jansson E. Effects of a one-year physical activity programme for women with systemic lupus erythematosus - a randomized controlled study. Lupus. 2016;25(6):602-16.
59. Perandini LA, Sales-de-Oliveira D, Almeida DC, Azevedo H, Moreira-Filho CA, Cenedeze MA, et al. Effects of acute aerobic exercise on leukocyte inflammatory gene expression in systemic lupus erythematosus. Exercise Immunology Review. 2016;22:64-81.
60. O'Dwyer T, Durcan L, Wilson F. Exercise and physical activity in systemic lupus erythematosus: A systematic review with meta-analyses. Seminars in Arthritis & Rheumatism. 2017;47(2):204-15.
61. Wu ML, Yu KH, Tsai JC. The Effectiveness of Exercise in Adults With Systemic Lupus Erythematosus: A Systematic Review and Meta-Analysis to Guide Evidence-Based Practice. Worldviews on Evidence-Based Nursing. 2017;14(4):306-15.
62. Benatti FB, Miyake CNH, Dantas WS, Zambelli VO, Shinjo SK, Pereira RMR, et al. Exercise Increases Insulin Sensitivity and Skeletal Muscle AMPK Expression in Systemic Lupus Erythematosus: A Randomized Controlled Trial. Frontiers in Immunology. 2018;9:906.
63. Middleton KR, Haaz Moonaz S, Hasni SA, Magana Lopez M, Tataw-Ayuketah G, Farmer N, et al. Yoga for systemic lupus erythematosus (SLE): Clinician experiences and qualitative perspectives from students and yoga instructors living with SLE. Complementary Therapies in Medicine. 2018;41:111-7.
64. Soriano-Maldonado A, Morillas-de-Laguno P, Sabio JM, Gavilan-Carrera B, Rosales-Castillo A, Montalban-Mendez C, et al. Effects of 12-week Aerobic Exercise on Arterial Stiffness, Inflammation, and Cardiorespiratory Fitness in Women with Systemic LUPUS Erythematosus: Non-Randomized Controlled Trial. Journal of Clinical Medicine. 2018;7(12):24.
65. Timoteo RP, Silva AF, Micheli DC, Candido Murta EF, Freire M, Teodoro RB, et al. Increased flexibility, pain reduction and unaltered levels of IL-10 and CD11b + lymphocytes in patients with systemic lupus erythematosus were associated with kinesiotherapy. Lupus. 2018;27(7):1159-68.
66. da Hora TC, Lima K, Maciel R. The effect of therapies on the quality of life of patients with systemic lupus erythematosus: a meta-analysis of randomized trials. Advances in Rheumatology. 2019;59(1):34.
67. Sheikh SZ, Kaufman K, Gordon BB, Hicks S, Love A, Walker J, et al. Evaluation of the self-directed format of Walk With Ease in patients with systemic lupus erythematosus: the Walk-SLE Pilot Study. Lupus. 2019;28(6):764-70.
68. Wu ML, Tsai JC, Yu KH, Chen JJ. Effects of physical activity counselling in women with systemic lupus erythematosus: A randomized controlled trial. International Journal of Nursing Practice. 2019;25(5):e12770.
69. Gavilan-Carrera B, Vargas-Hitos JA, Morillas-de-Laguno P, Rosales-Castillo A, Sola-Rodriguez S, Callejas-Rubio JL, et al. Effects of 12-week aerobic exercise on patient-reported outcomes in women with systemic lupus erythematosus. Disability & Rehabilitation. 2020:1-9.
70. Keramiotou K, Anagnostou C, Kataxaki E, Galanos A, Sfikakis PP, Tektonidou MG. The impact of upper limb exercise on function, daily activities and quality of life in systemic lupus erythematosus: a pilot randomised controlled trial. RMD Open. 2020;6(1):01.
71. Dionello CF, Souza PL, Rosa PV, Santana A, Marchon R, Morel DS, et al. Acute Neuromuscular Responses to Whole-Body Vibration of Systemic Lupus Erythematosus Individuals: A Randomized Pilot Study. Applied Sciences-Basel. 2021;11(1).
72. Kao VP, Wen HJ, Pan YJ, Pai CS, Tsai ST, Su KY. Combined aerobic and resistance training improves physical and executive functions in women with systemic lupus erythematosus. Lupus. 2021;30(6):946-55.
73. Lopes-Souza P, Dionello CF, Bernardes-Oliveira CL, Moreira-Marconi E, Marchon RM, Teixeira-Silva Y, et al. Effects of 12-week whole-body vibration exercise on fatigue, functional ability and quality of life in women with systemic lupus erythematosus: A randomized controlled trial. Journal of Bodywork and Movement Therapies. 2021;27:191-9.
74. Lu MC, Koo M. Effects of Exercise Intervention on Health-Related Quality of Life in Patients with Systemic Lupus Erythematosus: A Systematic Review and Meta-Analysis of Controlled Trials. Healthcare (Basel). 2021;9(9).
75. Patterson SL, Trupin L, Yazdany J, Dall'Era M, Lanata C, Dequattro K, et al. Physical Inactivity Independently Predicts Incident Depression in a Multi-Racial/Ethnic Systemic Lupus Cohort. Arthritis care & research. 2021;9:09.
76. Hashemi S, Habibagahi Z, Heidari M, Abdollahpour-Alitappeh M, Karimi MH. Effects of combined aerobic and anaerobic exercise training on cytokine profiles in patients with systemic lupus erythematosus (SLE); a randomized controlled trial. Transpl Immunol. 2022;70:101516.
77. Frade S, O'Neill S, Walsh S, Campbell C, Greene D, Bird SP, et al. Telehealth-supervised exercise in systemic lupus erythematosus: A pilot study. Lupus. 2023;32(4):508-20.
78. Lin MC, Livneh H, Lu MC, Chang CH, Chen ML, Tsai TY. Effects of a walking exercise programme on disease activity, sleep quality, and quality of life in systemic lupus erythematosus patients. Int J Nurs Pract. 2023;29(6):e13174.
79. Wohland H, Aringer M, Leuchten N. Physical exercise is associated with less fatigue, less pain and better sleep in patients with systemic lupus erythematosus. Clin Exp Rheumatol. 2023.
80. Shah M, Kavanaugh A, Coyle Y, Adams-Huet B, Lipsky PE. Effect of a culturally sensitive cholesterol lowering diet program on lipid and lipoproteins, body weight, nutrient intakes, and quality of life in patients with systemic lupus erythematosus. Journal of Rheumatology. 2002;29(10):2122-8.
81. Minami Y, Sasaki T, Arai Y, Kurisu Y, Hisamichi S. Diet and systemic lupus erythematosus: a 4 year prospective study of Japanese patients. Journal of Rheumatology. 2003;30(4):747-54.
82. Duffy EM, Meenagh GK, McMillan SA, Strain JJ, Hannigan BM, Bell AL. The clinical effect of dietary supplementation with omega-3 fish oils and/or copper in systemic lupus erythematosus. Journal of Rheumatology. 2004;31(8):1551-6.
83. Shah M, Adams-Huet B, Kavanaugh A, Coyle Y, Lipsky P. Nutrient intake and diet quality in patients with systemic lupus erythematosus on a culturally sensitive cholesterol lowering dietary program. Journal of Rheumatology. 2004;31(1):71-5.
84. Aghdassi E, Morrison S, Landolt-Marticorena C, Su J, Pineau CA, Gladman D, et al. The use of micronutrient supplements is not associated with better quality of life and disease activity in Canadian patients with systemic lupus erythematosus. J Rheumatol. 2010;37(1):87-90.

85. Minami Y, Hirabayashi Y, Nagata C, Ishii T, Harigae H, Sasaki T. Intakes of vitamin B6 and dietary fiber and clinical course of systemic lupus erythematosus: a prospective study of Japanese female patients. Journal of Epidemiology. 2011;21(4):246-54.
86. Davies RJ, Lomer MC, Yeo SI, Avloniti K, Sangle SR, D'Cruz DP. Weight loss and improvements in fatigue in systemic lupus erythematosus: a controlled trial of a low glycaemic index diet versus a calorie restricted diet in patients treated with corticosteroids. Lupus. 2012;21(6):649-55.
87. Elkan AC, Anania C, Gustafsson T, Jogestrand T, Hafstrom I, Frostegard J. Diet and fatty acid pattern among patients with SLE: associations with disease activity, blood lipids and atherosclerosis. Lupus. 2012;21(13):1405-11.
88. Khajehdehi P, Zanjaninejad B, Aflaki E, Nazarinia M, Azad F, Malekmakan L, et al. Oral supplementation of turmeric decreases proteinuria, hematuria, and systolic blood pressure in patients suffering from relapsing or refractory lupus nephritis: a randomized and placebo-controlled study. Journal of Renal Nutrition. 2012;22(1):50-7.
89. Everett ST, Wolf R, Contento I, Haiduc V, Richey M, Erkan D. Short-term patient-centered nutrition counseling impacts weight and nutrient intake in patients with systemic lupus erythematosus. Lupus. 2015;24(12):1321-6.
90. Shamekhi Z, Amani R, Habibagahi Z, Namjoyan F, Ghadiri A, Saki Malehi A. A Randomized, Double-blind, Placebo-controlled Clinical Trial Examining the Effects of Green Tea Extract on Systemic Lupus Erythematosus Disease Activity and Quality of Life. Phytotherapy Research. 2017;31(7):1063-71.
91. Rothman D, Khan F, Rudin V. Individualized Diet and Lifestyle Modifications Reverse Symptoms of Systemic Lupus Erythematosus. Journal of Medical Internet Research. 2018;20(9):21-.
92. Pocovi-Gerardino G, Correa-Rodriguez M, Callejas-Rubio JL, Rios-Fernandez R, Martin-Amada M, Cruz-Caparros MG, et al. Beneficial effect of Mediterranean diet on disease activity and cardiovascular risk in systemic lupus erythematosus patients: a cross-sectional study. Rheumatology. 2021;60(1):160-9.
93. Gwinnutt JM, Wieczorek M, Rodríguez-Carrio J, Balanescu A, Bischoff-Ferrari HA, Boonen A, et al. Effects of diet on the outcomes of rheumatic and musculoskeletal diseases (RMDs): systematic review and meta-analyses informing the 2021 EULAR recommendations for lifestyle improvements in people with RMDs. RMD Open. 2022;8(2).
94. Knippenberg A, Robinson GA, Wincup C, Ciurtin C, Jury EC, Kalea AZ. Plant-based dietary changes may improve symptoms in patients with systemic lupus erythematosus. Lupus. 2022;31(1):65-76.
95. Stege H, Budde MA, Grether-Beck S, Krutmann J. Evaluation of the capacity of sunscreens to photoprotect lupus erythematosus patients by employing the photoprovocation test. Photodermatology, Photoimmunology & Photomedicine. 2000;16(6):256-9.
96. Herzinger T, Plewig G, Rocken M. Use of sunscreens to protect against ultraviolet-induced lupus erythematosus. Arthritis & Rheumatism. 2004;50(9):3045-6.
97. Zahn S, Graef M, Patsinakidis N, Landmann A, Surber C, Wenzel J, et al. Ultraviolet light protection by a sunscreen prevents interferon-driven skin inflammation in cutaneous lupus erythematosus. Experimental Dermatology. 2014;23(7):516-8.
98. Squance ML, Reeves G, Attia J, Bridgman H, Guest M. Self-reported Lupus flare: Association with everyday home and personal product exposure. Toxicology Reports. 2015;2:880-8.
99. Xu D, You X, Wang Z, Zeng Q, Xu J, Jiang L, et al. Chinese Systemic Lupus Erythematosus Treatment and Research Group Registry VI: Effect of Cigarette Smoking on the Clinical Phenotype of Chinese Patients with Systemic Lupus Erythematosus. PLoS ONE [Electronic Resource]. 2015;10(8):e0134451.
100. Abdul Kadir WD, Jamil A, Shaharir SS, Md Nor N, Abdul Gafor AH. Photoprotection awareness and practices among patients with systemic lupus erythematosus and its association with disease activity and severity. Lupus. 2018;27(8):1287-95.
101. Raymond WD, Hamdorf M, Furfaro M, Eilertsen GO, Nossent JC. Smoking associates with increased BAFF and decreased interferon-γ levels in patients with systemic lupus erythematosus. Lupus Sci Med. 2021;8(1).
102. Li X, He L, Wang J, Wang M. Illness uncertainty, social support, and coping mode in hospitalized patients with systemic lupus erythematosus in a hospital in Shaanxi, China. PLoS ONE [Electronic Resource]. 2019;14(2):e0211313.
103. (Editor) AE, (Editor) MZ. JBI Manual for Evidence Synthesis JBI2020 [Available from: https://synthesismanual.jbi.global.
104. Howick J, Chalmers I, Glasziou P, Greenhalgh T, Heneghan C, Liberati, et al. The Oxford Levels of Evidence 2 2011 [Available from: https://www.cebm.ox.ac.uk/resources/levels-of-evidence/ocebm-levels-of-evidence.
